# Supplementary material for: TM4SF19-mediated control of lysosomal activity in macrophages contributes to obesity-induced inflammation and metabolic dysfunction
Source: Nat Commun. 2024 Mar 30;15:2779. doi: 10.1038/s41467-024-47108-8 (PMC10981689; doi:10.1038/s41467-024-47108-8)

Supplementary Figures and Figure Legends

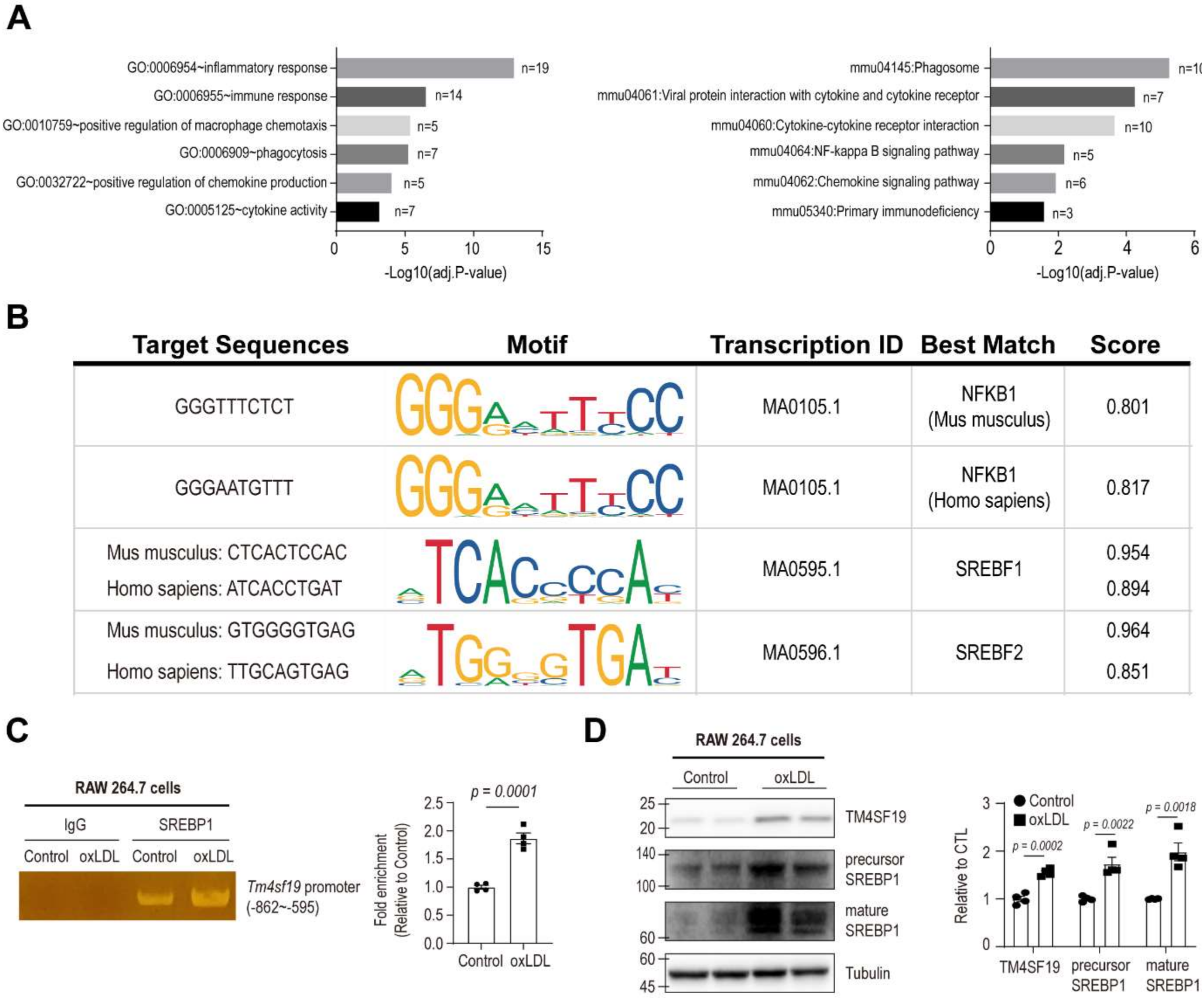

Figure S1. Characterization of obesity-induced upregulation of *Tm4sf19* in adipose tissue macrophages.

A. Gene ontology (GO) enrichment analysis and KEGG pathway analysis of the top 100 genes upregulated by obesity from each dataset.

B. Conserved sequences of NFKB1, SREBF1, and SREBF2 binding sites in the promoter of mouse *Tm4sf19* and human *TM4SF19*. Scores described the similarity values of the transcription factor binding sites.

C. Chromatin immunoprecipitation (ChIP) enrichment analysis of recruitment of SREBP1 to *Tm4sf19* promoter in RAW264.7 cells treated with oxLDL (100 µg/ml for 24 hours) by PCR and q-PCR. PCR amplification was carried out with DNA fragments immunoprecipitated by anti-SREBP1 antibody and negative control IgG (n = 4 biologically independent replicates).

D. Immunoblot analysis of TM4SF19 and SREBP1 protein levels in RAW264.7 cells after oxLDL treatment (100 µg/ml) for 24 hours.

Source data are provided as a Source Data file.

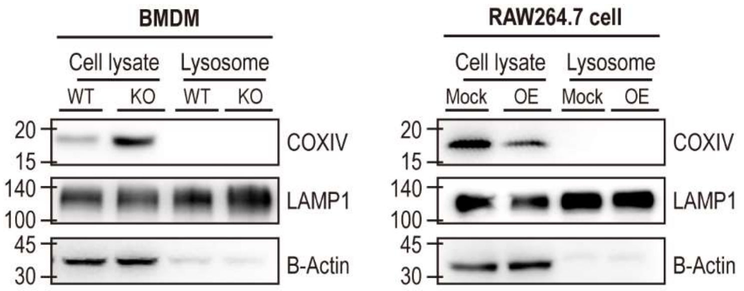

Figure S2. Immunoblot analysis of lysosome fraction from TM4SF19 overexpressing RAW264.7 cells and TM4SF19 KO BMDMs.

Immunoblot analysis confirmed the purity of lysosomal fraction obtained from BMDM of WT and TM4SF19 KO mice, and TM4SF19 overexpressing RAW 246.7 cells used for Figure 2E. LAMP1 was used as a lysosomal marker and β-actin was used as a cytosol marker. (n = 3 biologically independent replicates).

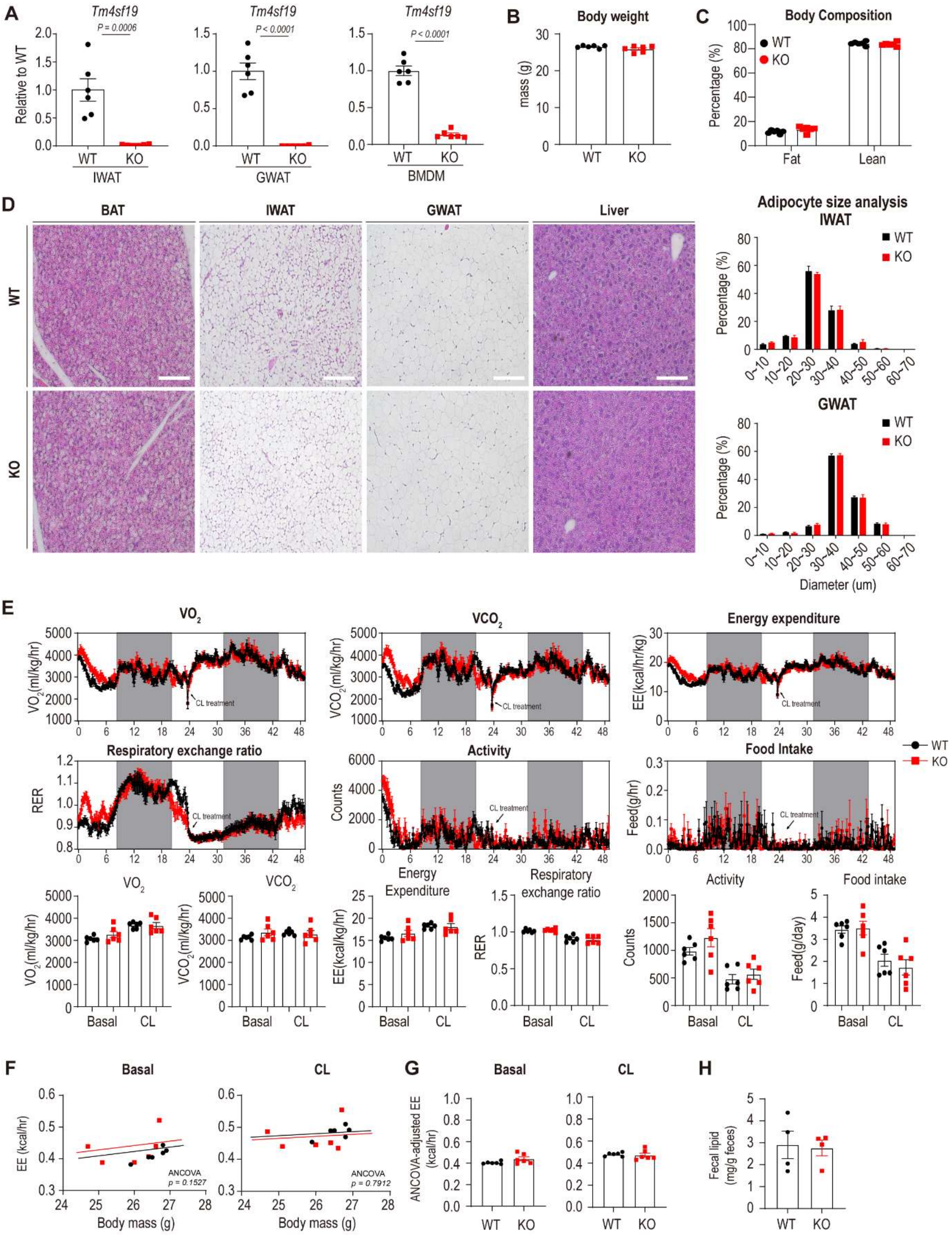

Figure S3. Metabolic phenotype analysis in WT and TM4SF19 KO mice under chow diet feeding.

A. *Tm4sf19* mRNA expression levels in IWAT, GWAT, and bone-marrow-derived macrophages (BMDM) of wild-type (WT) and TM4SF19 KO mice fed a normal chow diet (NCD) (n = 6

biologically independent replicates).
B. Body weight monitoring of WT and TM4SF19 KO mice for 10 weeks (n = 10 mice).
C. Body composition analysis of WT and TM4SF19 KO mice (n = 9 mice).
D. Representative hematoxylin and eosin (H&E) stained image of paraffin sections of BAT, IWAT, GWAT, and liver, along with quantification of lipid droplet sizes in IWAT and GWAT from WT and TM4SF19 KO mice (n = 6 mice). Scale bar = 50μm
E. Indirect calorimetry analysis of WT and TM4SF19 KO mice before and after CL 316,243 treatment (1mg/kg, i.p.) (n = 6 mice). The points of CL treatment are indicated by the arrows in the graph.
F. Regression plots of energy expenditure against body mass and ANCOVA test using body mass as a covariate. (n = 6 mice).
G. ANCOVA -adjusted energy expenditure (EE) (predicted at the mean body mass (26.2 g)). (n = 6 mice).
H. Lipid content in feces of WT and TM4SF19 KO mice (n = 4 mice).
Source data are provided as a Source Data file.

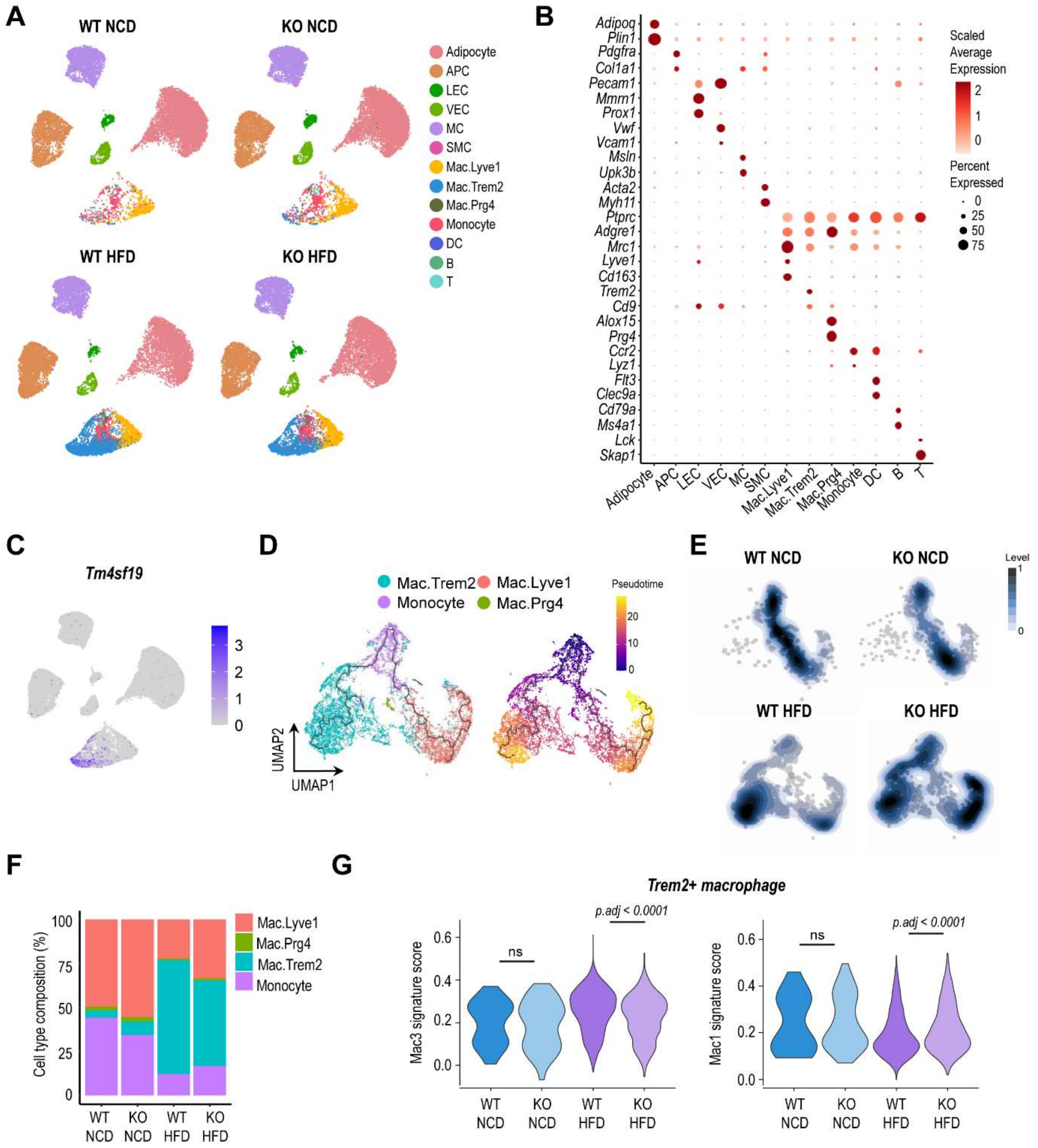

Figure S4. Characterization of cell types in mouse GWAT and trajectory analysis of monocytes/macrophages.

A. UMAP plots isolated from GWAT of NCD and HFD-fed WT and TM4SF19 KO mice.

B. Dot plot showing the marker gene expression to define the cell types.

C. *Tm4sf19* gene expression on UMAP plot. The density of purple colors presents the expression levels of *Tm4sf19* in GWAT.

D. UMAP plot showing the differentiation paths from monocyte to macrophages constructed using Monocle3. Cells were colored by cell types (left) and pseudotime (right). The line on the UMAP plot shows a differentiation trajectory.

E. UMAP plots split by condition (WT NCD, TM4SF19 KO NCD, WT HFD, TM4SF19 KO

HFD). The color indicates the density of cells in each condition.
F. Cell type compositions of monocyte and macrophage population in each condition (WT NCD, TM4SF19 KO NCD, WT HFD, TM4SF19 KO HFD).
G. Mac3 and Mac1 signature scores of *Trem2*+ macrophage in each condition (WT NCD, TM4SF19 KO NCD, WT HFD, TM4SF19 KO HFD).
Source data are provided as a Source Data file.

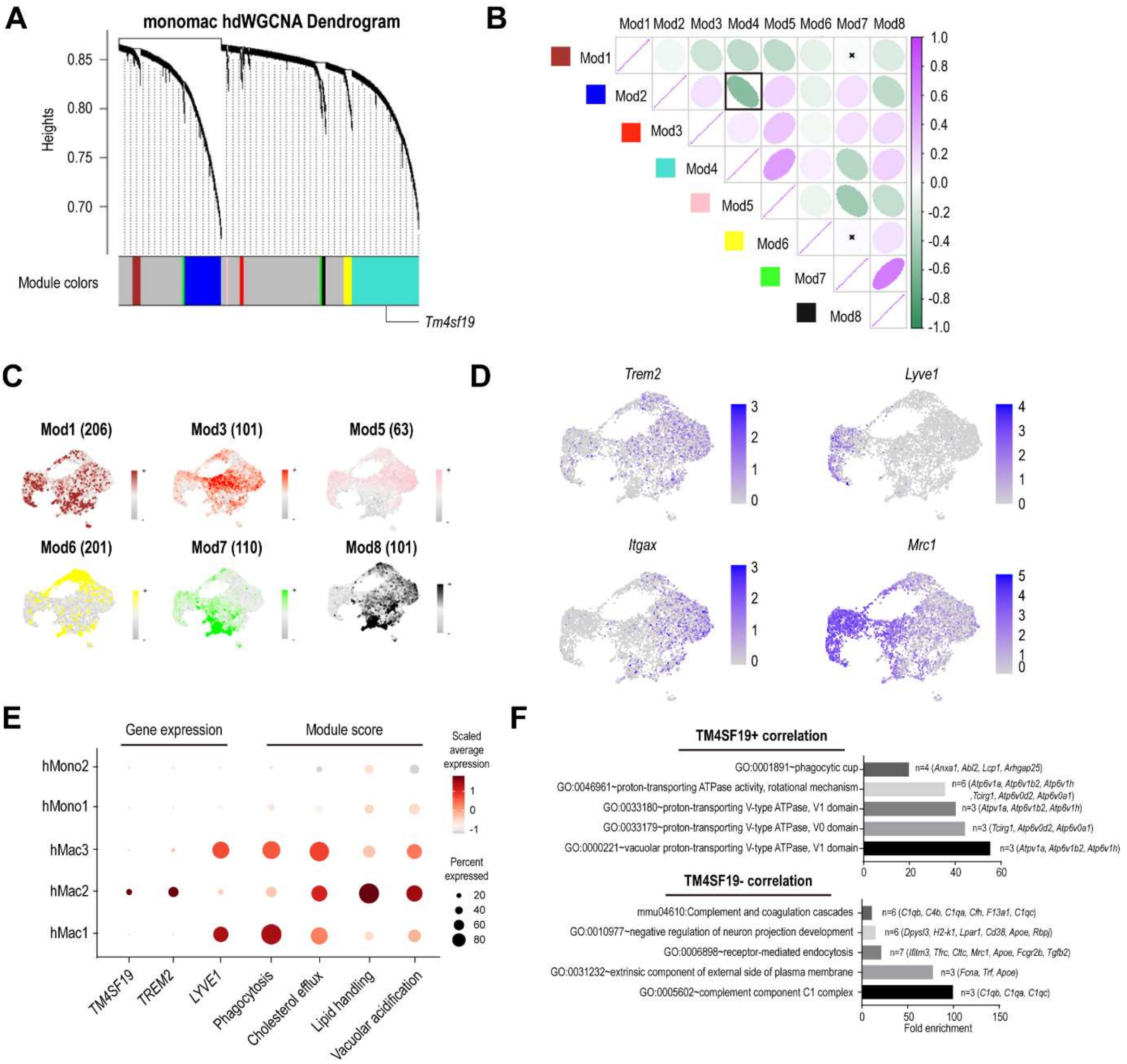

Figure S5. Molecular characterization of adipose tissue macrophage subtypes by snRNA-seq analysis.

A. WGCNA dendrogram showing co-expression modules from the network analysis of monocyte and macrophage population from WT HFD condition. Each leaf represents a gene, and each color indicates a co-expression gene module. Genes expressed over 5% of cells were used for analysis.

B. Correlation between each module from A. *Tm4sf19* gene is included in Mod4.

C. UMAP plot showing module eigengenes (MEs) score. The number indicates the number of genes in each module in the WT HFD mice.

D. *Trem2*, *Lyve1*, *Itgax*, *Mrc1* gene expression on UMAP plot. The density of purple colors presents the expression levels of each gene in GWAT of WT HFD.

E. Dot plot showing specific gene expression and module scores in human macrophage and monocyte population (GSE176171).

F. Gene ontology enrichment analysis of different gene expressions of TM4SF19 positive/negative correlation genes in monocyte/macrophage clusters in WT HFD groups.

Source data are provided as a Source Data file.

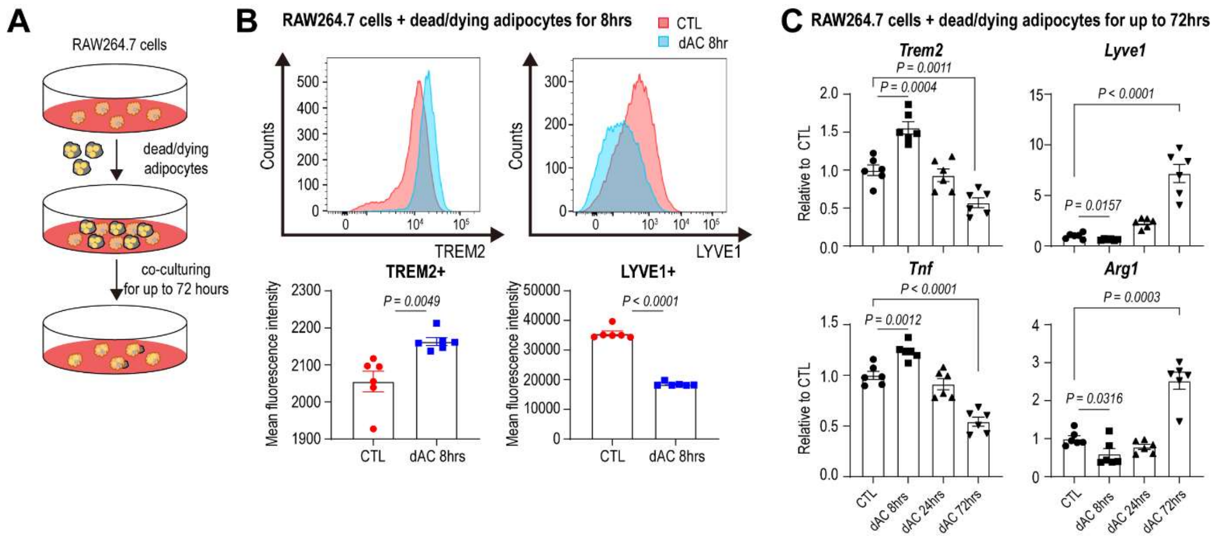

Figure S6. Molecular characterization of macrophages by single-cell analysis and macrophage/adipocyte co-cultures

A. Schematic diagram of RAW264.7 cells co-cultured with dead/dying adipocytes.

B. Histogram of TREM2+ and LYVE1+ macrophages in RAW264.7 cells co-cultured with dead/dying adipocytes for 8 hours (n = 6 biologically independent replicates).

C. qPCR analysis of *Trem2*, *Lyve1*, *Tnf*, and *Arg1* gene expression levels in RAW264.7 cells co-cultured with dead/dying adipocytes for up to 72 hours (n = 6 biologically independent replicates).

Source data are provided as a Source Data file.

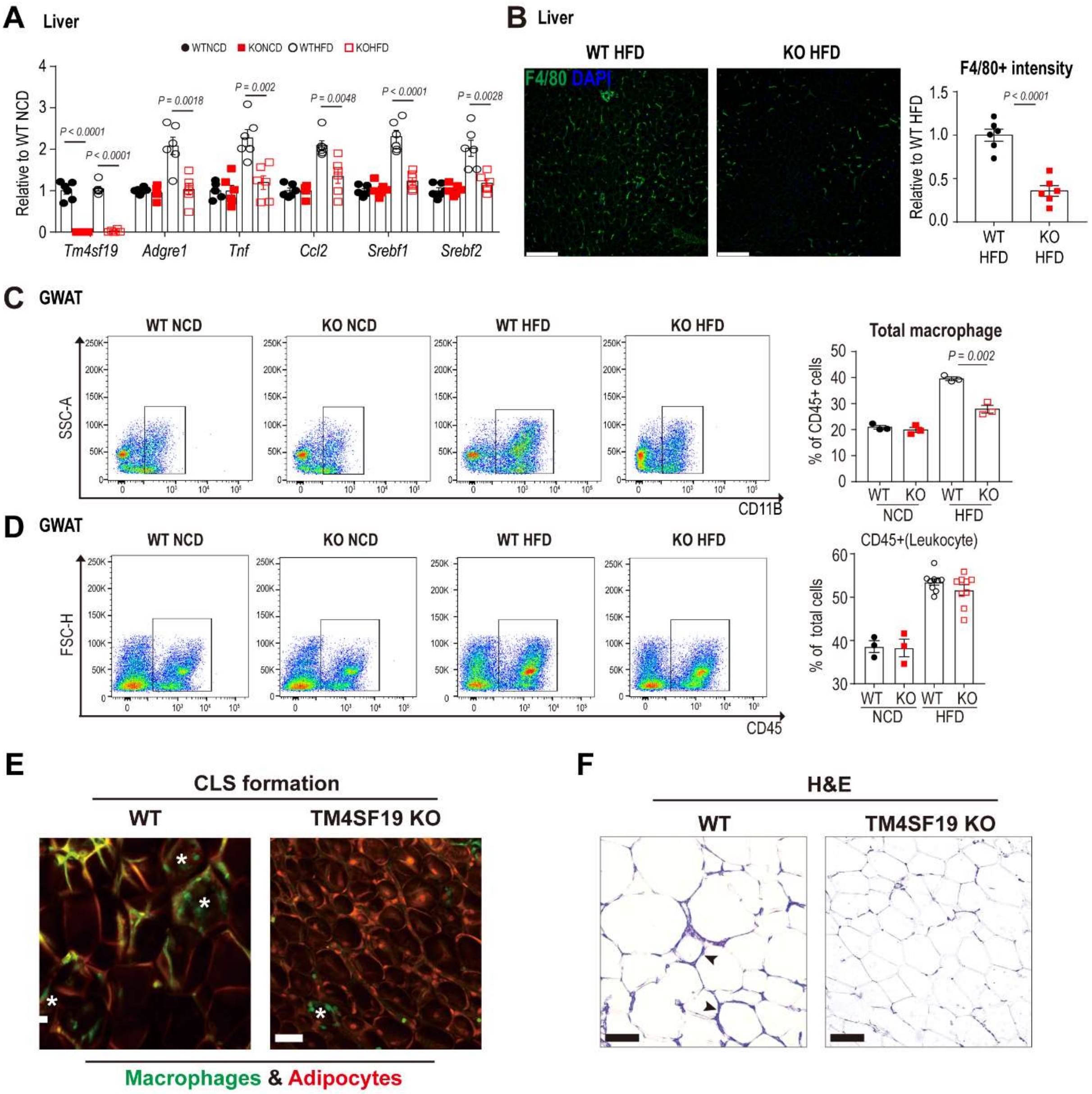

Figure S7. Effects of TM4SF19 KO on HFD-induced macrophage recruitment in liver and adipose tissue

A. mRNA expression levels of genes involved in inflammatory response and lipogenesis in liver of WT and TM4SF19 KO HFD mice (n = 6 mice).

B. Immunofluorescence staining of F4/80 in paraffin sections from liver of WT and TM4SF19 KO HFD mice (n = 6). Scale bar = 50 μm

C-D. Representative flow profiles of CD11B (C) and CD45 (D) expression levels from GWAT of WT and TM4SF19 KO mice fed NCD or HFD for 12 weeks (n = 3, 9 mice).

E. Representative fluorescent images of CX3CR1-GFP expression (visualized monocytes/macrophages) and Pdgfra-Cre/Td-tomato expression (visualized PDGFRA+ cell-derived progeny (adipocytes and PDGFRA+ cells)) in GWAT of WT and TM4SF19 KO mice.

F. Representative hematoxylin and eosin (H&E) stained images of paraffin sections of GWAT of WT and TM4SF19 KO mice. White asterisks (E) and black arrows (F) indicated the crown-like structures (CLS). Scale bars = 50 μm.

Source data are provided as a Source Data file.

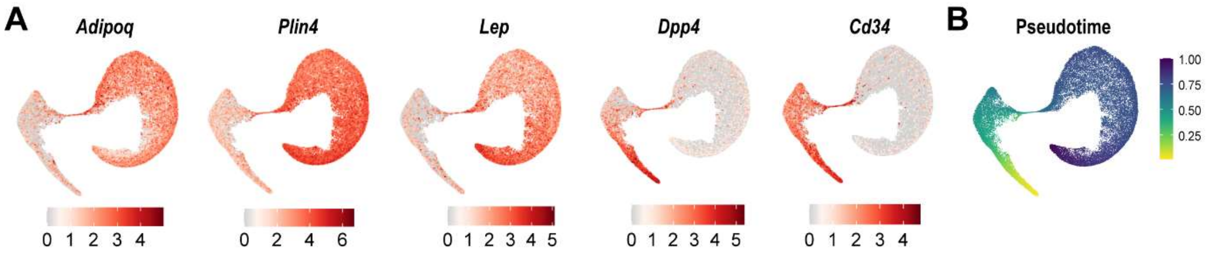

Figure S8. Trajectory analysis of APC/Adipocyte populations
A. *Adipoq*, *Plin4*, *Lep*, *Dpp4*, and *Cd34* gene expression in APCs and Adipocyte populations.
B. Pseudotime showing differentiation trajectory of APCs to adipocytes.

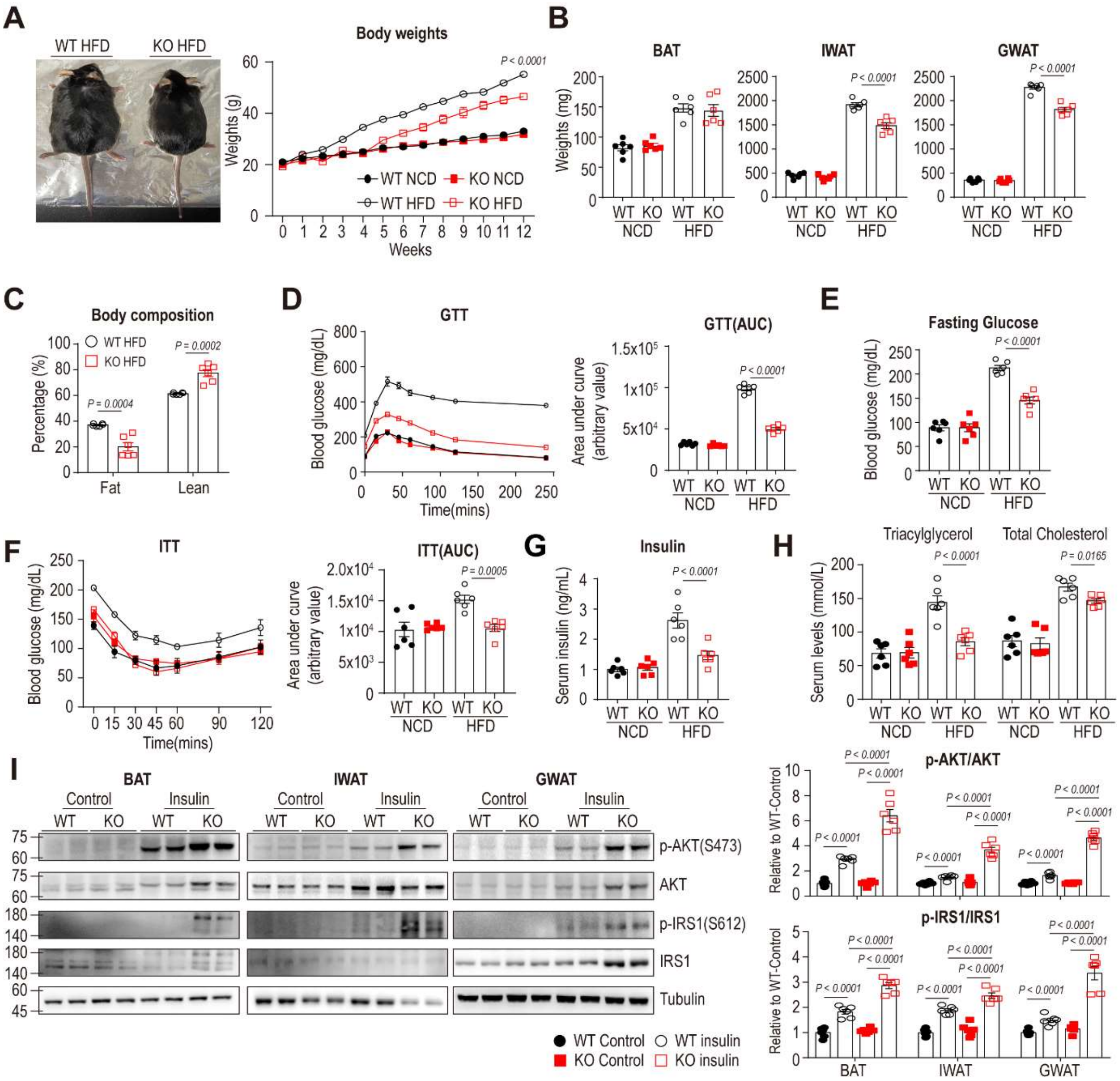

Figure S9. TM4SF19 KO protects mice from obesity-induced metabolic dysfunction.

A. Representative images of wild-type (WT) and TM4SF19 KO mice after 12 weeks of high-fat diet (HFD) feeding, and mouse weight monitoring of WT and TM4SF19 KO mice fed a normal chow diet (NCD) or HFD for 12 weeks (n = 6 mice).

B-F. Adipose tissue mass (B), body composition (C), glucose tolerance test (GTT) (D), fasting glucose levels (E), and insulin tolerance test (ITT) (F) of WT and TM4SF19 KO mice after 12 weeks of NCD and HFD feeding (n = 6 mice).

G. Serum insulin levels of WT and TM4SF19 KO mice after 12 weeks NCD and HFD feeding (n = 6).

H. Serum triacylglycerol and total cholesterol of WT and TM4SF19 KO mice after 12 weeks of NCD and HFD feeding (n = 6 mice).

I. Immunoblot analysis of p-AKT, AKT, p-IRS1, and IRS1 protein expression levels of WT and TM4SF19 KO mice after 12 weeks of HFD feeding. Tissues were collected 10 min after insulin treatment (0.75IU/kg) (n = 6 mice).

Source data are provided as a Source Data file.

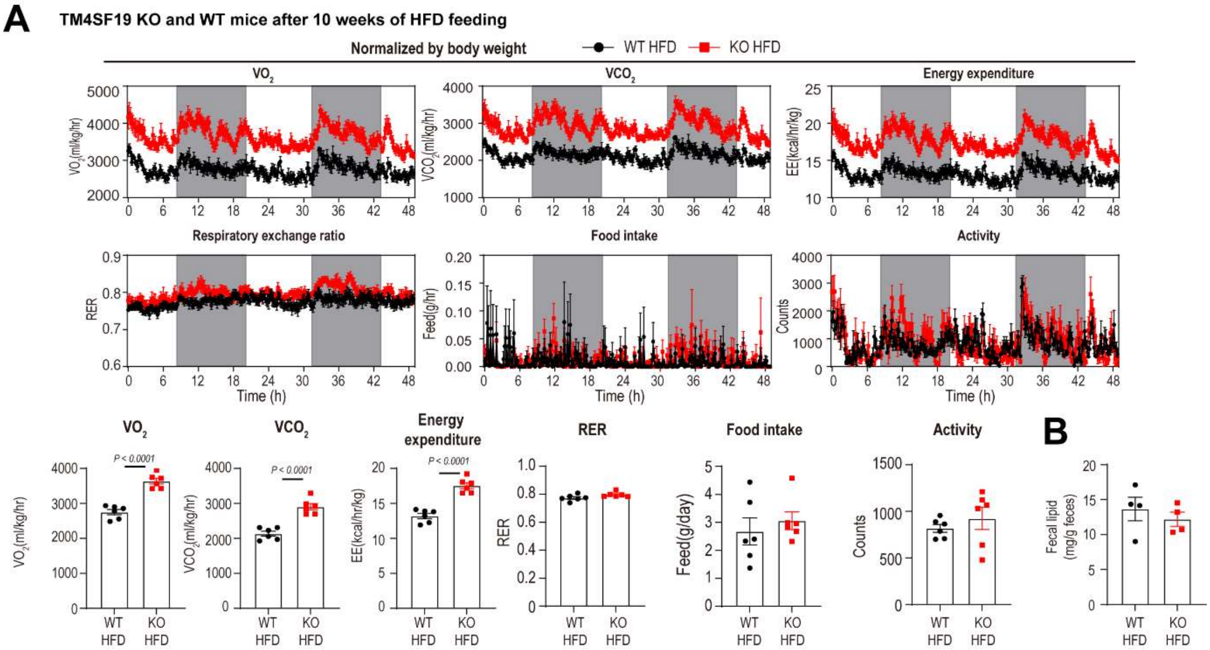

Figure S10. Indirect calorimetry analysis of HFD-fed TM4SF19 KO and WT mice.

A. Indirect calorimetry analysis of WT and TM4SF19 KO mice after 10 weeks of HFD feeding (n = 6 mice). VO_2, VCO_2, and energy expenditure normalized by body weight.

B. Lipid content in feces of WT and TM4SF19 KO mice after 10 weeks of HFD feeding (n = 4 mice).

Source data are provided as a Source Data file.

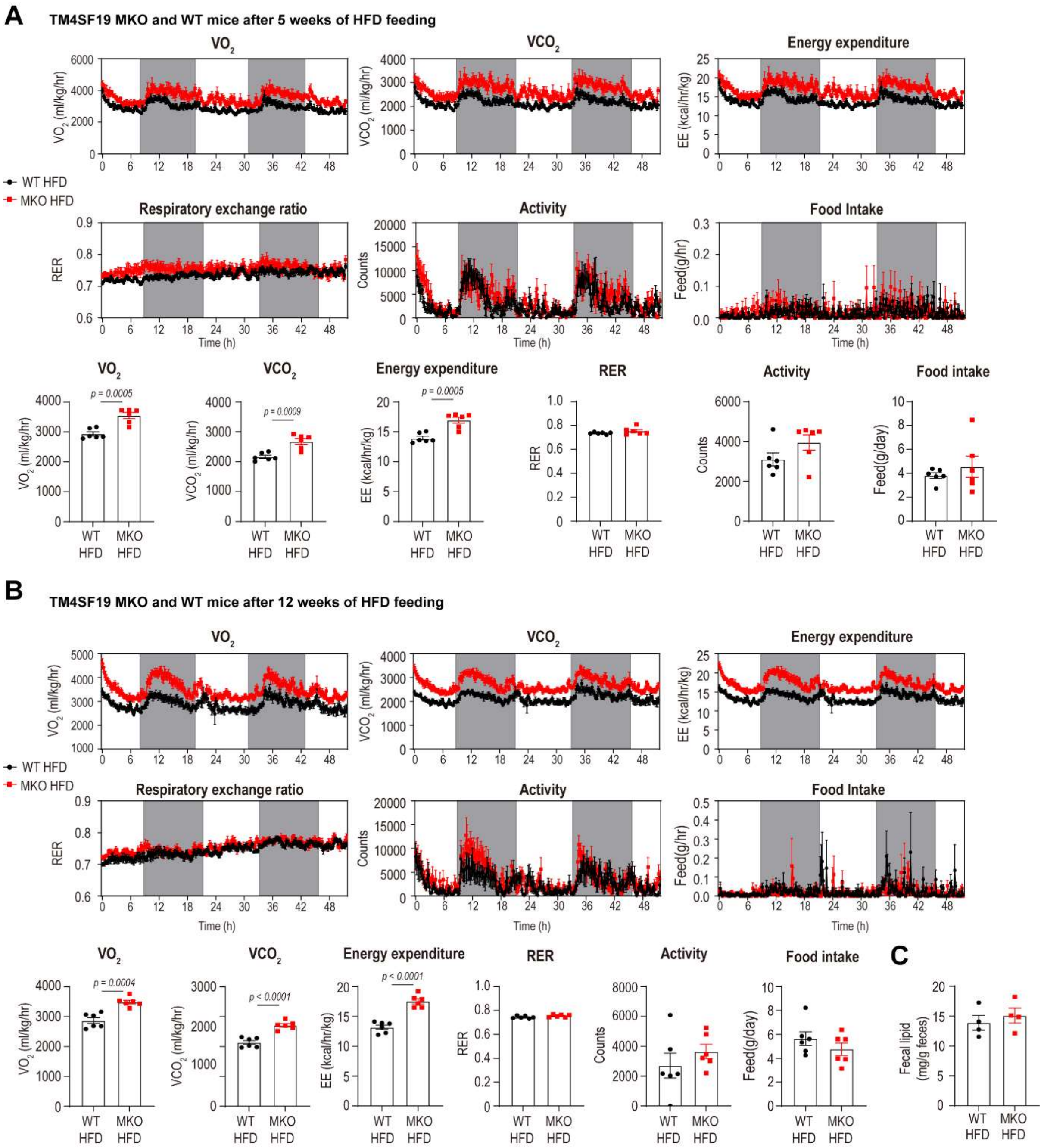

Figure S11. Indirect calorimetry analysis of TM4SF19 MKO and WT mice.

A-B. Indirect calorimetry analysis of WT and macrophage-specific TM4SF19 MKO mice after 5 weeks (A) and 12 weeks (B) of HFD feeding (n = 6 mice). VO_2, VCO_2, and energy expenditure were normalized by body weight (n = 6 mice).

B. Lipid content in feces of WT and TM4SF19 MKO mice after 12 weeks of HFD feeding (n = 4 mice).

Source data are provided as a Source Data file.

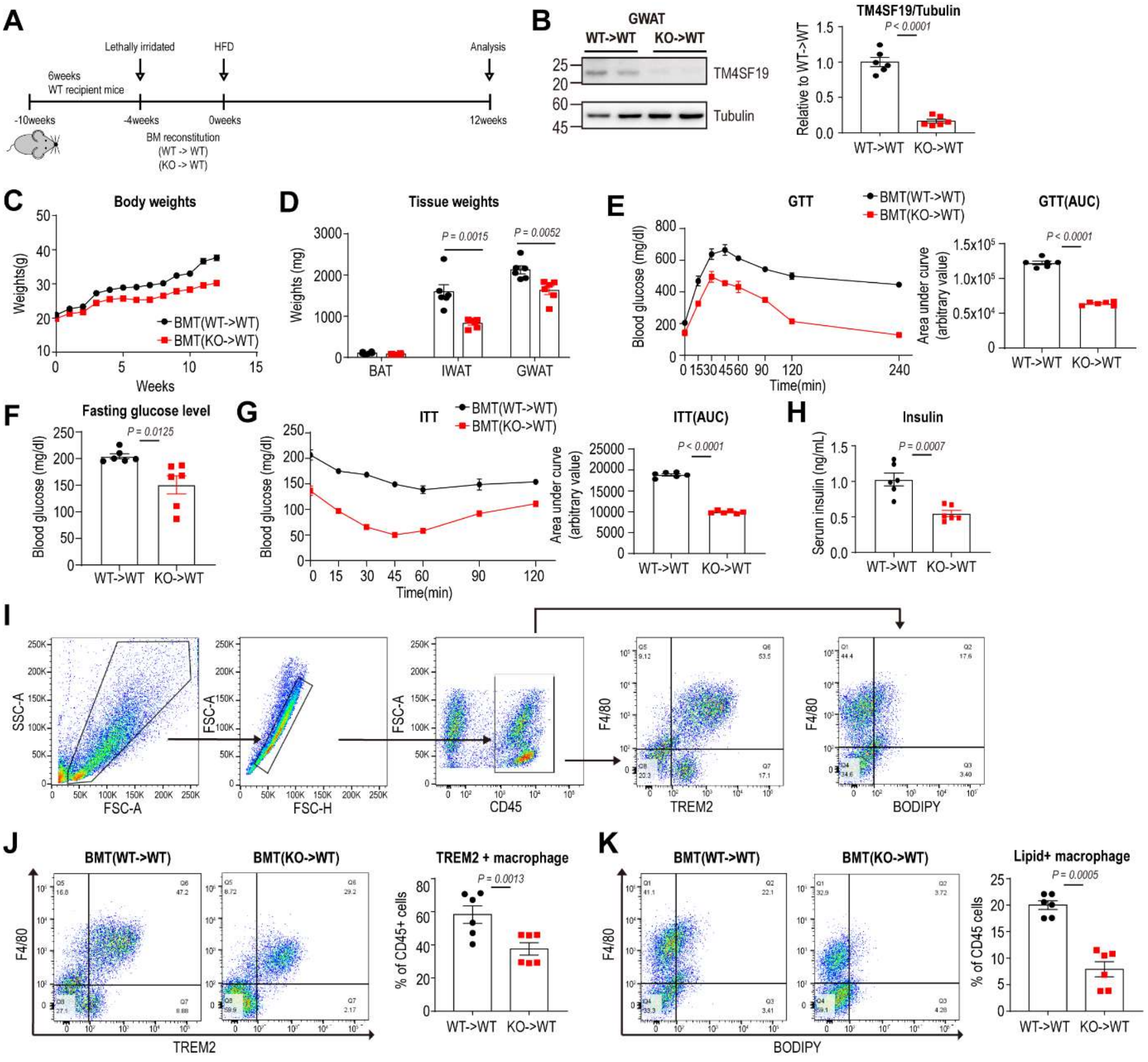

Figure S12. Bone marrow transplantation of TM4SF19 KO cells reduced adiposity and improved obesity-related metabolic dysfunction.

A. Experimental strategy of bone marrow transplantation (BMT) of TM4SF19 KO cells. After bone marrow (BM) reconstitution, HFD feeding was begun for 12 weeks.

B. Immunoblot analysis of TM4SF19 protein in GWAT of BMT KO mice after HFD feeding (n = 6 mice).

(C-D) Body weights monitoring (C) and adipose tissue weights (D) of BMT KO mice after HFD feeding (n = 6 mice).

(E-G) Glucose tolerance test (GTT) (E), fasting glucose levels (F) and insulin tolerance test (ITT) (G) of BMT KO mice after 12 weeks of HFD feeding (n = 6 mice).

H. Serum insulin levels of BMT WT and KO mice after HFD feeding (n = 6 mice).

(I-K). Representative flow profiles of the gating strategy (I), TREM2 positive macrophages (J) and lipid-associated macrophages (K) from GWAT of BMT WT and KO mice fed a HFD for 12 weeks (n = 6 mice).

Source data are provided as a Source Data file.

Supplementary Table S1. Top 100 upregulated genes from different publicly available transcriptomic analyses related to Figure. 1.

GSE150102 Top 100 upregulated genes		
**Gene.symbol**	**LogFC**	**adj.P.Val**
Gpr50	7.890476556	0.001007
Slc5a7	7.141463377	0.000254
Tm4sf19	6.978878633	0.000216
Tph2	6.900754589	0.000121
Itgad	6.892271013	0.001814
Mstn	6.506231466	0.000014
Cck	6.440078236	0.000375
Trdn	6.401707161	0.000125
S100a7l2	6.084438949	0.001061
Atp6v0d2	5.989930696	0.000192
Olr1	5.938936996	0.000168
Wif1	5.919110142	0.000931
Lipf	5.812053067	0.000301
Vsig8	5.777637934	0.000354
Rgs1	5.67091904	0.000266
Gpnmb	5.667433221	0.000085
Eef1a2	5.622523292	0.000041
Otop1	5.61908277	0.000087
Pex5l	5.566735059	0.000041
Gdf3	5.482301852	0.000696
Mmp12	5.458365177	0.000285
Ubd	5.422495296	0.000723
Hephl1	5.346107514	0.001476
Grid1	5.305010534	0.000316
Il1rn	5.224092193	0.000117
Serpinb9b	5.18116133	0.000422
Gm5150	5.114769885	0.000907
Myh15	5.110145099	0.041507
Dppa3	5.049513422	0.000230
Hmga2	5.046214068	0.009796
Serpinb1c	5.034865355	0.001767
Lipn	5.034706329	0.000041
Dsg1a	4.847153531	0.006625

Dsg1b	4.834303364	0.000721
Spp1	4.715826833	0.012169
Tfr2	4.696939778	0.000040
Siglec15	4.671861703	0.002069
Npy	4.629758461	0.000157
Xirp2	4.600211522	0.000013
Ppp1r3a	4.591795883	0.001103
Brinp2	4.591011408	0.000339
Adam8	4.525814818	0.000203
Ltf	4.455184488	0.045771
St18	4.43578045	0.001942
Dlgap2	4.373301168	0.000069
F7	4.347670427	0.000071
Pvalb	4.341038627	0.000403
Il7r	4.33908119	0.000230
Gzme	4.336507219	0.000021
Slc37a2	4.315773452	0.000173
Npas4	4.267222561	0.000085
Sncb	4.186794065	0.001144
Fgf13	4.176235694	0.000318
Itgax	4.163303609	0.000175
Apoc4	4.111802467	0.004131
Areg	4.096051051	0.003092
Hapln4	4.086582287	0.001291
Nptx1	4.08111701	0.005071
S100a8	4.078727216	0.000187
Ear1	4.067183446	0.000462
Rhov	4.05157647	0.039607
Chil3	4.030903099	0.000771
Plppr4	4.027572067	0.000036
Mrc2	4.026833459	0.000014
Mmp3	4.026159053	0.000216
Dnmt3l	3.970974616	0.000358
Col10a1	3.959037251	0.000173
Mmp8	3.943842765	0.000627
Fosl1	3.931283441	0.000514
Nxf7	3.912193139	0.000813
Clec4d	3.90088119	0.000576

Trem2	3.89713253	0.000441
Slc15a5	3.888149863	0.001008
Acoxl	3.876354686	0.000625
Cngb3	3.864682202	0.002768
Ptprn	3.841263973	0.003865
Ceacam19	3.836246697	0.001004
Arl14epl	3.830183902	0.017945
Oit3	3.828538741	0.002343
Tlr13	3.790451969	0.000274
Ina	3.749649729	0.001713
Ereg	3.742668918	0.002979
Ankdd1a	3.72638737	0.002219
Mmp13	3.682757317	0.000613
H2bc7	3.663369797	0.000129
Slc15a3	3.635776038	0.000413
Car12	3.635765918	0.000352
Fbxo40	3.635224043	0.000230
Cdkn1a	3.630344167	0.000042
Gdf15	3.628152951	0.002276
Tnip3	3.626565868	0.000383
Olfr1388	3.603100238	0.007750
Dppa2	3.601949713	0.000037
Cyp2b9	3.577586218	0.026157
Ccl3	3.566283775	0.001007
Fitm1	3.562296488	0.026499
Pla2g4e	3.559056842	0.001712
Sirpb1c	3.527554229	0.000216
Igsf21	3.521337949	0.000230
Oxtr	3.500335459	0.000089

**GSE59034 Top 100 upregulated genes**		
**Gene.symbol**	**LogFC**	**adj.P.Val**
EGFL6	3.15	0.0001700
SPP1	2.77	0.0000124
TDRD6	2.62	0.0000070
TM4SF19	2.38	0.0000258

MMP9	2.16	0.0000124
MSR1	2.14	0.0000195
CHI3L1	1.97	0.0003350
MSC	1.94	0.0000036
STMN2	1.92	0.0005790
C3AR1	1.79	0.0000587
FCGR1CP	1.76	0.0000124
IFI30	1.75	0.0000173
IL1RN	1.73	0.0000934
ABCC3	1.7	0.0000043
FPR3	1.68	0.0000930
TNC	1.68	0.0000037
CD163	1.67	0.0000266
MS4A6E	1.65	0.0000454
/FCGR1B	1.63	0.0000032
SELE	1.61	0.0001650
FCGR1A	1.61	0.0000033
IGHD	1.57	0.0071500
C1orf204	1.56	0.0000064
ALCAM	1.55	0.0000589
VSIG4	1.55	0.0000401
LCP1	1.55	0.0000070
AQP9	1.54	0.0004760
LBP	1.54	0.0000169
IGSF6	1.53	0.0000359
CCL18	1.51	0.0030100
MRC1	1.51	0.0001090
MRC1	1.51	0.0001090
FCGR2A	1.51	0.0000206
HP	1.5	0.0013100
JCHAIN	1.49	0.0045200
ITGB2	1.48	0.0000165
CCR1	1.47	0.0000065
ACP5	1.46	0.0000286
HLA-DRB1	1.45	0.0597000
DHRS9	1.45	0.0000128
NCKAP1L	1.44	0.0000527
SLCO2B1	1.44	0.0000384

CD180	1.44	0.0000061
CHIT1	1.43	0.0015800
KYNU	1.43	0.0000046
NPL	1.41	0.0000051
RGS1	1.4	0.0007380
CYBB	1.38	0.0000620
GPR183	1.38	0.0000511
TFEC	1.38	0.0000069
ITGAX	1.37	0.0000699
C1orf162	1.37	0.0000154
VNN1	1.36	0.0000046
IGLJ3	1.35	0.0142000
DCSTAMP	1.34	0.0001440
UCHL1	1.34	0.0000431
ST14	1.34	0.0000155
PLEK	1.34	0.0000118
IL6	1.33	0.0005110
CD84	1.33	0.0000725
MS4A14	1.3	0.0004570
FCER1G	1.3	0.0000905
CD53	1.3	0.0000339
RNASE6	1.3	0.0000098
MS4A4A	1.29	0.0004000
FCGBP	1.29	0.0000203
MMP7	1.28	0.0005690
TREM2	1.27	0.0003490
C1QB	1.27	0.0002510
CD209	1.27	0.0000294
NCEH1	1.26	0.0000260
CXorf21	1.26	0.0000228
ALPL	1.26	0.0000086
TIGAR	1.26	0.0000013
LYZ	1.25	0.0005640
AOAH	1.25	0.0001310
PRG4	1.24	0.0005440
CCL2	1.24	0.0003540
KRR1	1.24	0.0000186
FCGR2C	1.23	0.0001430

IL18	1.23	0.0000239
HLA-DRB5	1.22	0.0110000
C1QC	1.22	0.0007500
MXRA5	1.22	0.0001680
IGHG1	1.21	0.0183000
FCGR2B	1.21	0.0001250
CTSS	1.21	0.0001230
CP	1.2	0.0009980
CCL19	1.2	0.0000896
C14orf99	1.19	0.0088500
CD52	1.19	0.0003810
F13A1	1.19	0.0002830
MILR1	1.19	0.0001070
DOCK2	1.19	0.0000509
CXCL16	1.19	0.0000215
NCF2	1.18	0.0000743
CECR1	1.18	0.0000434
P2RX6	1.18	0.0000036
PTPRC	1.17	0.0001710
CD28	1.17	0.0000111

**GSE182930 Top 100 upregulated genes**		
**Gene.symbol**	**LogFC**	**adj.P.Val**
Spp1	6.625957971	0.0133000
Sfrp5	6.458689959	0.0014000
Cxcl2	5.248440106	0.0436000
Atp6v0d2	4.765768962	0.0130000
Il1rn	4.726162268	0.0010500
Thbs1	4.692036743	0.0019700
Gpnmb	4.479968789	0.0114000
S100a8	4.422122606	0.0320000
F7	4.329044855	0.0021200
Adam8	4.32237103	0.0030500
Timp1	4.304811014	0.0071100
Mmp12	4.164477499	0.0259000
Ubd	4.027556352	0.0016200

Rgs1	3.933677541	0.0047100
Ccl3	3.790841493	0.0273000
Dhrs9	3.735028861	0.0054100
Gp49a	3.729441998	0.0013400
Lilr4b	3.729441998	0.0013400
Pla2g7	3.727345821	0.0010500
Basp1	3.701524387	0.0010300
Cdkl4	3.66701496	0.0018700
Clec4d	3.644742763	0.0133000
Tm4sf19	3.63519044	0.0306000
Thbs2	3.633779725	0.0020500
Slc5a7	3.612987003	0.0365000
Slc15a3	3.604827237	0.0010300
Il7r	3.583291662	0.0069300
Saa3	3.563811565	0.0248000
Lilrb4a	3.549166814	0.0012500
Lilrb4	3.549166814	0.0012500
Gm5150	3.527920772	0.0025500
Ccl9	3.492332903	0.0012500
Lox	3.490037951	0.0068500
Slc37a2	3.469916882	0.0037400
C5ar1	3.454855718	0.0010500
Tlr13	3.442756315	0.0010300
Ccl2	3.432576539	0.0056500
Ctss	3.423687091	0.0010500
Cd300lb	3.417782188	0.0026800
Ccr1	3.387900059	0.0148000
Cd44	3.371281564	0.0010300
Nceh1	3.35337968	0.0038400
Trem2	3.320080192	0.0047600
Cd200r1	3.274735649	0.0029700
Ccl4	3.273540608	0.0391000
Cd300a	3.262257043	0.0020500
Atf3	3.262216889	0.0054100
Cd300c2	3.252913382	0.0013200
Cd84	3.240886299	0.0026000
C3ar1	3.231954638	0.0010300
Pvalb	3.189841599	0.0209000

Lgmn	3.18561272	0.0012500
Ms4a6d	3.184870593	0.0079000
Ccl6	3.178552165	0.0026600
Peg3	3.17323718	0.0021200
Itgax	3.172549319	0.0156000
Soat1	3.17017966	0.0020400
Pdk4	3.154163256	0.0027400
Hk3	3.152299148	0.0014600
Cd300ld	3.122031172	0.0015600
Sfrp4	3.103470306	0.0112000
Bmp3	3.102888781	0.0012500
Csf3r	3.101793076	0.0357000
Cd72	3.099539309	0.0012500
Mmp3	3.093383905	0.0018700
Lgals3	3.084939719	0.0056000
Bst1	3.072922016	0.0111000
Ccl7	3.07032996	0.0392000
Mpeg1	3.070166992	0.0022200
Dpep2	3.069268603	0.0024800
Cd68	3.068781206	0.0024000
Bcl2a1b	3.061579979	0.0010300
Tfpi2	3.054772239	0.0012500
Plek	3.052010885	0.0036500
Ccr5	3.046208034	0.0030800
Peg10	3.042379901	0.0099100
Crtac1	3.027525842	0.0016200
Msr1	3.023822418	0.0045900
Adgre1	3.023677678	0.0027300
Arl11	3.011574958	0.0043000
Pik3r5	2.99801592	0.0012500
Pik3ap1	2.983902685	0.0026300
F10	2.975985606	0.0036200
Arhgap25	2.975953864	0.0122000
Lcp1	2.974516853	0.0022500
Vnn1	2.970512459	0.0036200
Tlr1	2.964651212	0.0032500
Cd53	2.960755403	0.0013100
Adss1	2.957567127	0.0016200

Adssl1	2.957567127	0.0016200
Dock8	2.940010922	0.0042000
Slc11a1	2.916787163	0.0016200
Gpr35	2.913936377	0.0061100
Hspb7	2.912618016	0.0032600
Fcgr1	2.906637542	0.0107000
Itgb2	2.905744252	0.0027800
Myo1f	2.899386047	0.0037800
Rab7b	2.897457259	0.0019300
Pirb	2.895900874	0.0013100
Lilrb3	2.895900874	0.0013100

Supplementary Table S2 Population characteristics of human subcutaneous adipose tissue, related to Figure 5.

**Variable**	
Age, *y*	9 ~ 79
Male, *n* (%)	62.5
Female, *n* (%)	37.5
BMI, *kg/m²*	19.11 ~ 32.38
Height, *m*	138.6 ~ 178
Weight, *kg*	40.8 ~ 91.2

Supplementary Table S3 Primers used for genotyping and qPCR

Genotyping primers		
Name	Forward primer	Reverse primer
Tm4sf19 (C57BL/6N-Tm4sf19em1cyagen)	1. TTAGAGGAAGTCCTTGAGACCCC<br>2. ATCTGTATGACCGTTCACTTTGGA	AACATGTGGCCAACCTTTATGAGA
Tm4sf19 (C57BL/6JSmoc-Tm4sf19em1Smoc)	AGGGCAAAGAAGGAAGTGGCTAAT	CAGGAAGGGGGCAGACAAGGAGT
Csf1r-CreER (FVB-Tg (Csf1r-cre/Esr1*)1Jwp/J))	CTTCCAAAGCATGGTCCAGT	TGAACCAGCTCCCTATCTGC
qPCR primers		
Transcript	Forward primer	Reverse primer
Tm4sf19	CTTGCCATCACAAACAGGATGC	GAAAGAGGAGCGCGATGTTG
TM4SF19	GGATGTCACCTACCTGTTGAGG	ACTGAAGCAGCCGTATCTCCAG
Ccl2	CTGGATCGGAACCAAATGAG	AAGGCATCACAGTCCGAGTC
Adgre1	GTGACTCACCTTGTGGTCCT	CAGACACTCATCAACATCTGCG
Rgs1	AATGCAGTGGTCTCAGTCTCTGG	ATAGTCCTCACAAGCCAACCAGA
Msr1	GCTCACTTTGGACAAGGTACTG	GCTTAGTACTCCCCACTGGTT
Srebf1	CGACTACATCCGCTTCTTGCAG	CCTCCATAGACACATCTGTGCC
Srebf2	AGAAAGAGCGGTGGAGTCCTTG	GAACTGCTGGAGAATGGTGAGG
Tnf	GGTGCCTATGTCTCAGCCTCTT	GCCATAGAACTGATGAGAGGGAG
IRS1	AGTCTGTCGTCCAGTAGCACCA	ACTGGAGCCATACTCATCCGAG
INSR	GCAACATCACCCACTACCTGGT	GAATGGTGGAGACCAGGTCCTC
PDE3B	TCCTGGCTTACAGCAGATCCAC	GGCAGCCATAACTCTCATCAGG
LEP	GCTGTGCCCATCCAAAAAGTCC	CCCAGGAATGAAGTCCAAACCG
FASN	TTCTACGGCTCCACGCTCTTCC	GAAGAGTCTTCGTCAGCCAGGA

PDE4D	GGACACTTTGGAGGACAATCGTG	CCTTTTCCGTGTCTGACTCACC
NF-κB	GCAGCACTACTTCTTGACCACC	TCTGCTCCTGAGCATTGACGTC
Lyve1	TGGTGTTACTCCTCGCCTCT	TTCTGCGCTGACTCTACCTG

Uncropped western blot images used for supplementary figures

Figure S1D

TM4SF19

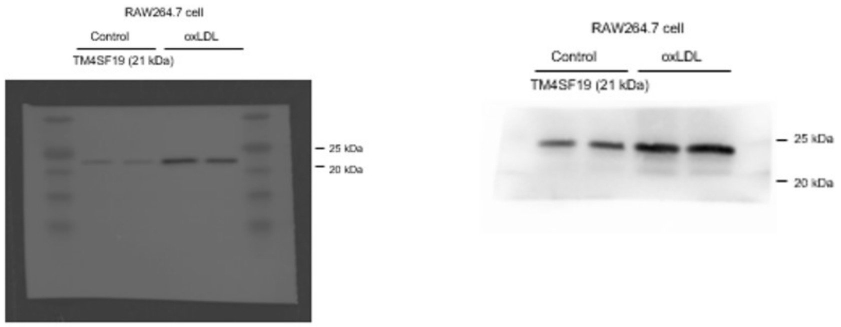

SREBP1

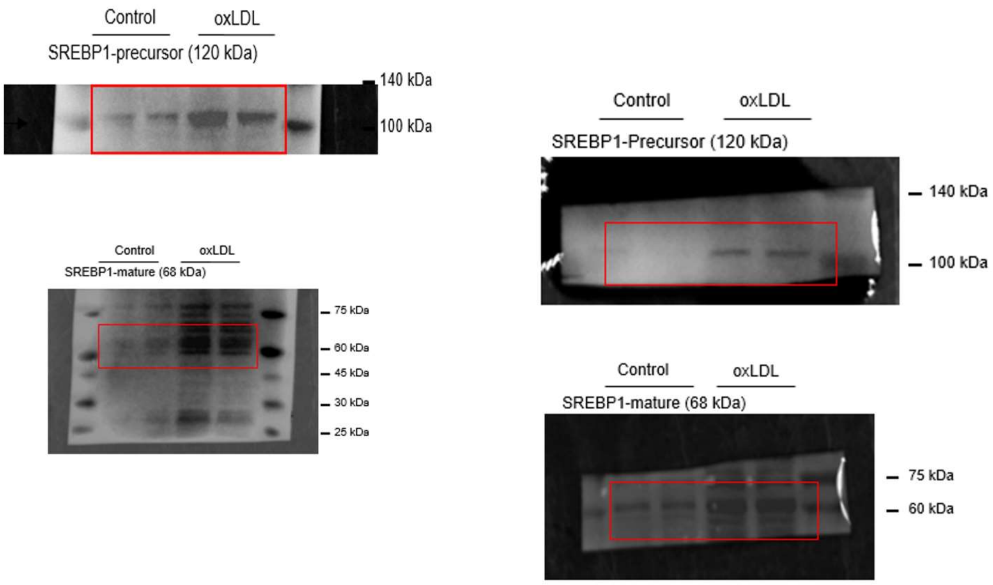

Tubulin

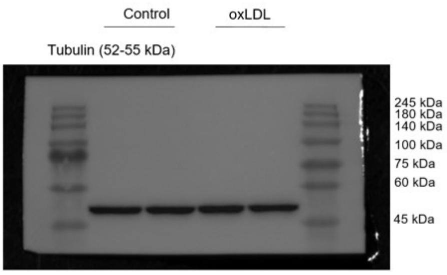

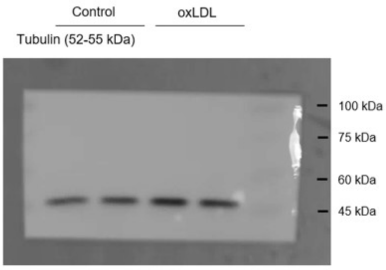

Figure S2 BMDM

Figure S2 BMDM

COXIV

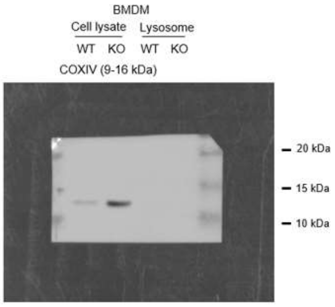

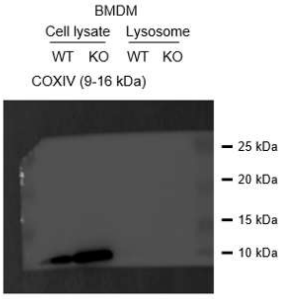

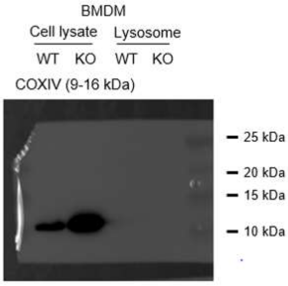

Figure S2 BMDM

LAMP1

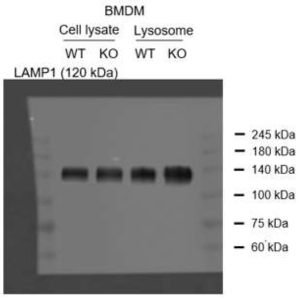

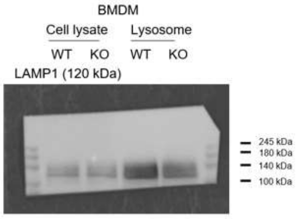

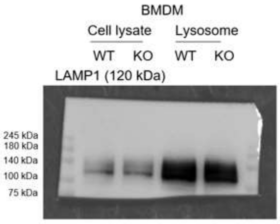

Figure S2 RAW264.7 cell

Figure S2 RAW264.7 cell

COXIV

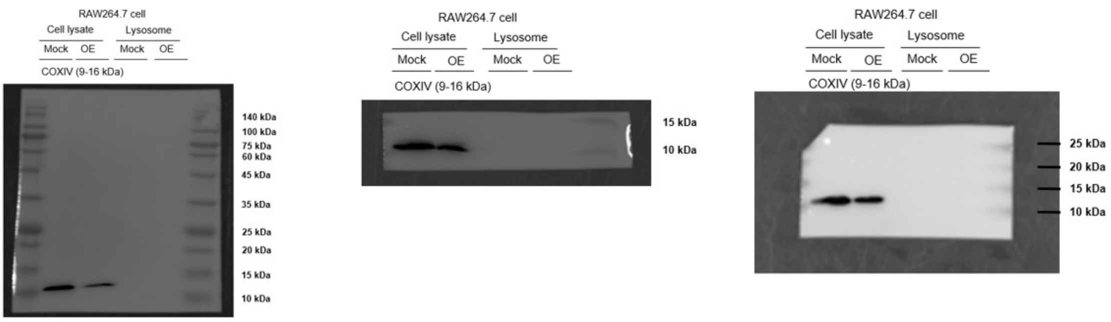

Figure S2 RAW264.7 cell

LAMP1

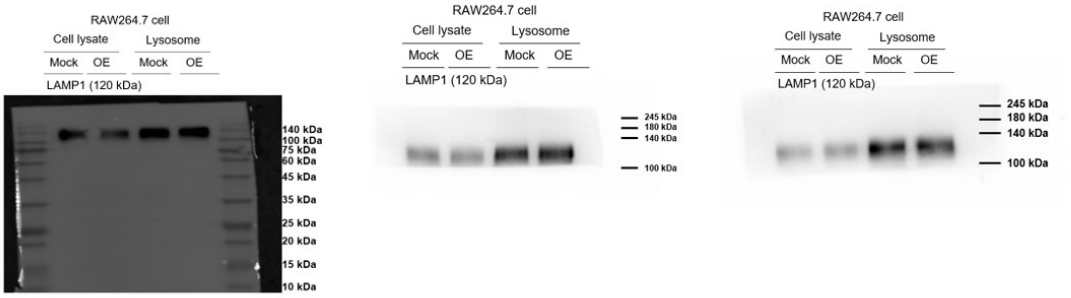

Figure S2 BMDM

B-Actin

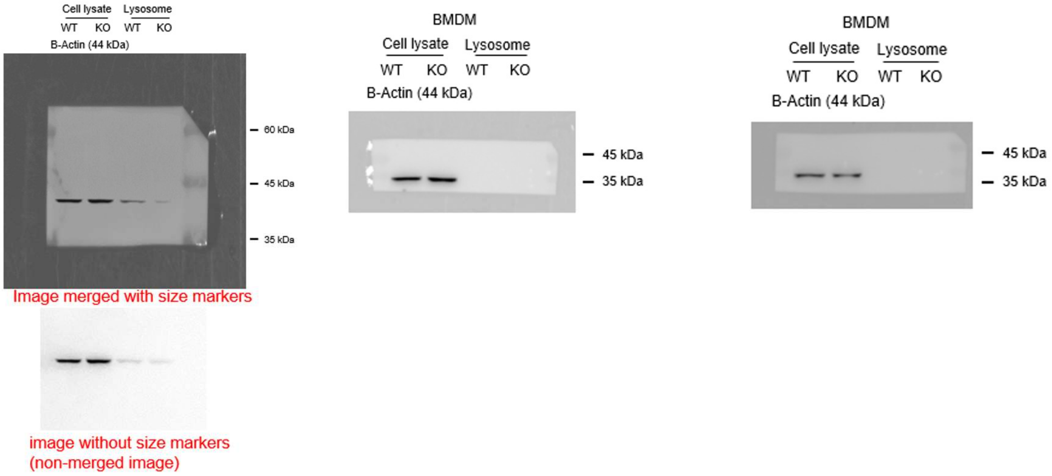

Figure S9I BAT

Figure S9I BAT

P-AKT

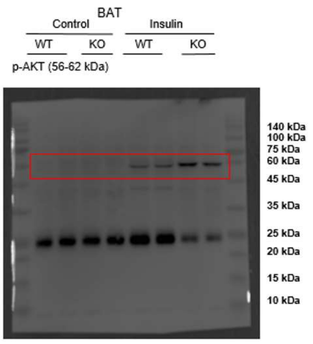

Image merged with size markers

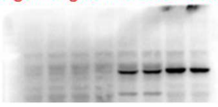

image without size markers
(non-merged image)

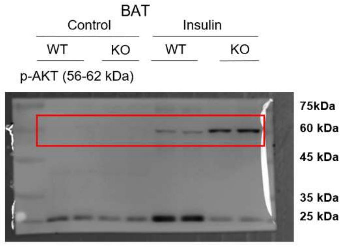

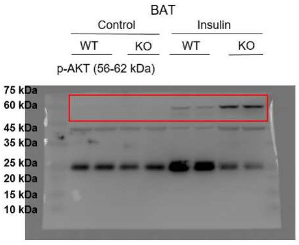

Figure S9I BAT

AKT

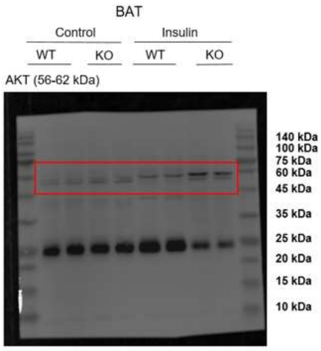

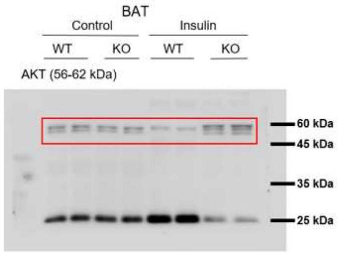

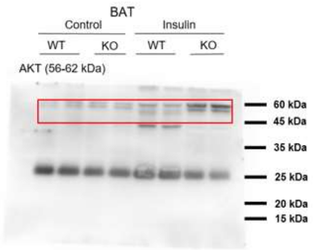

Figure S9I BAT

P-IRS1

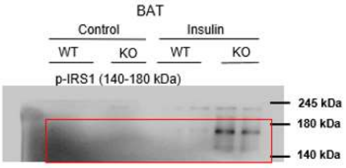

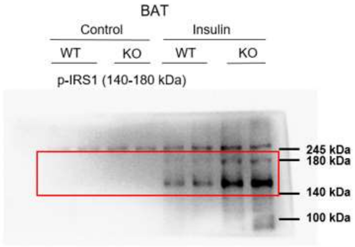

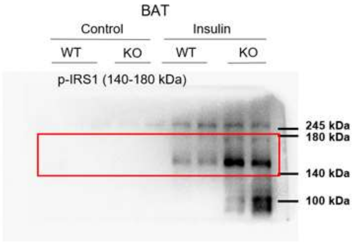

Figure S9I BAT

IRS1

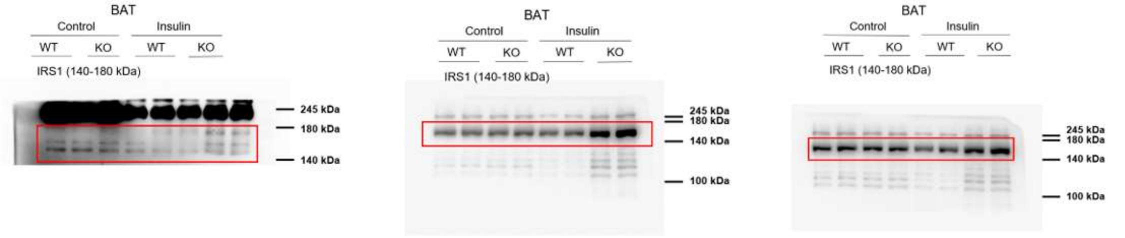

Figure S9I BAT

Tubulin

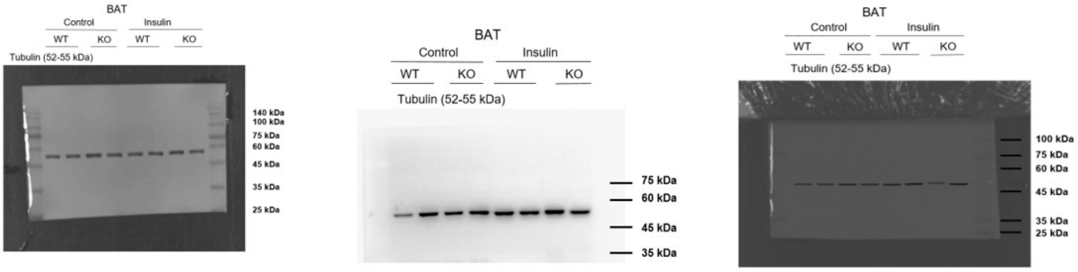

Figure S9I IWAT

Figure S9I IWAT

P-AKT

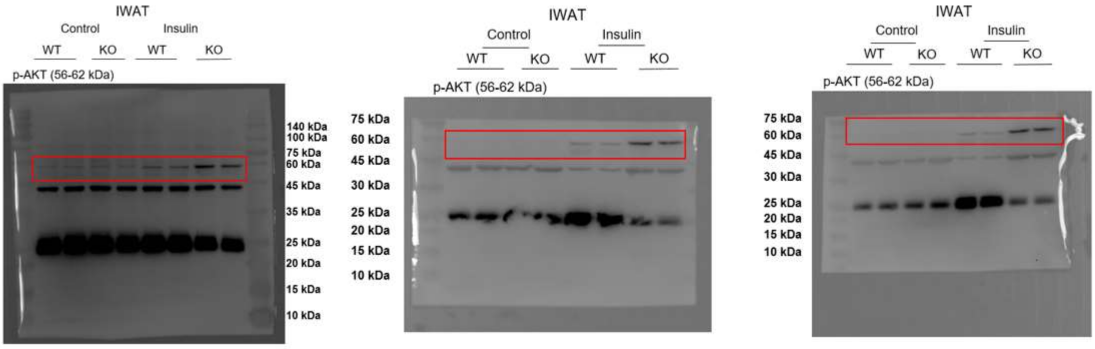

Figure S9I IWAT

AKT

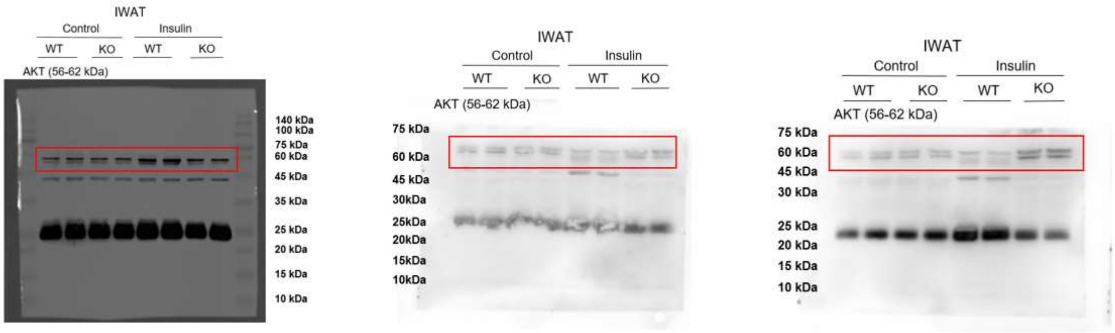

Figure S9I IWAT

P-IRS1

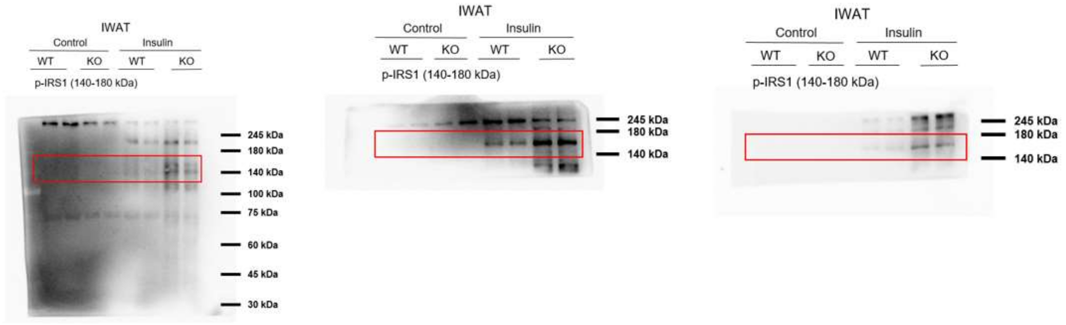

Figure S9I IWAT

IRS1

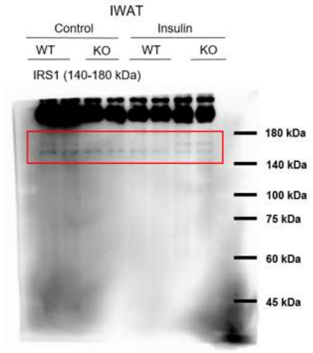

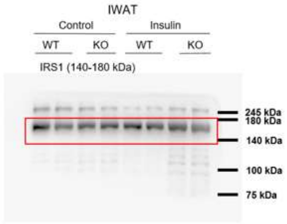

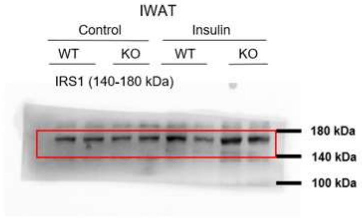

Figure S9I IWAT

Tubulin

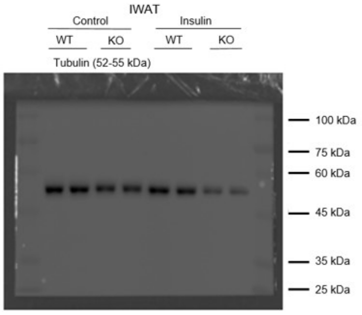

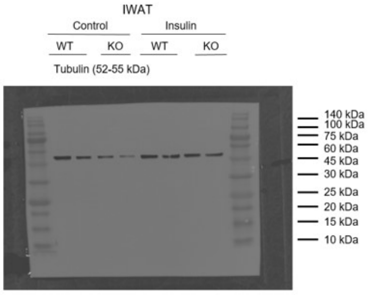

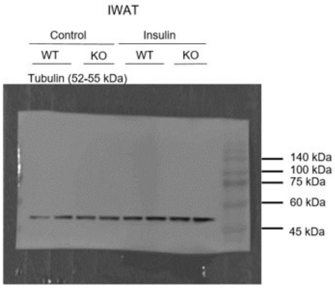

Figure S9I GWAT

Figure S9I GWAT

P-AKT

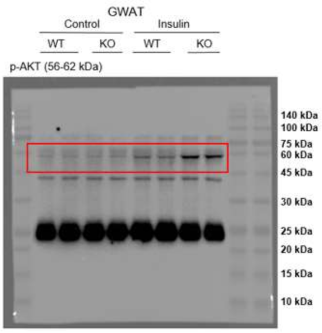

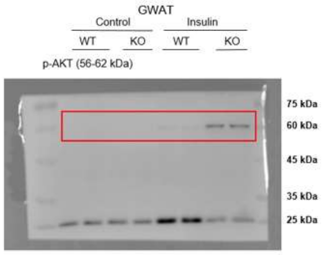

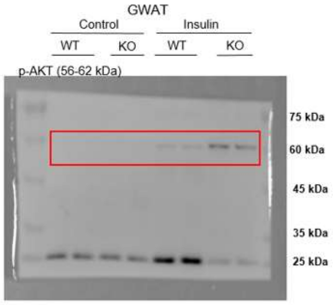

Figure S9I GWAT

AKT

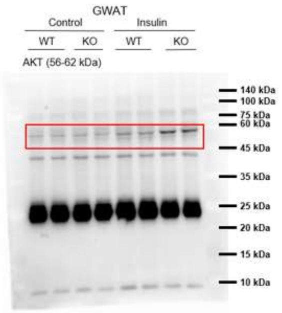

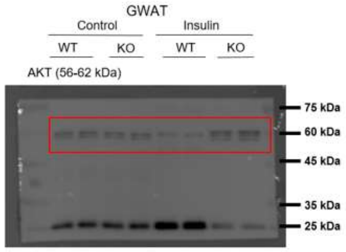

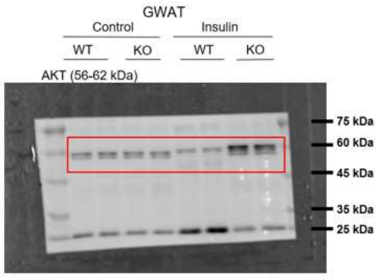

Figure S9I GWAT

P-IRS1

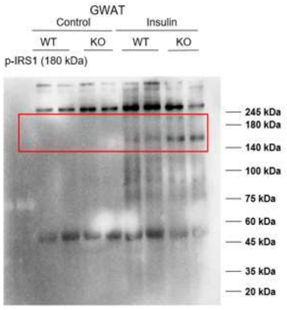

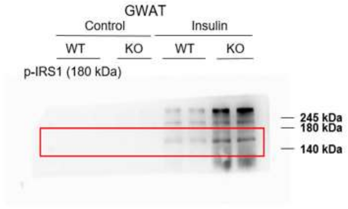

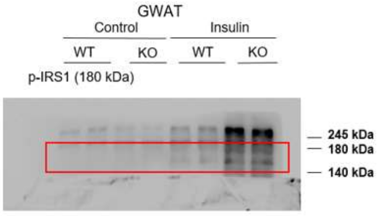

Figure S9I GWAT

IRS1

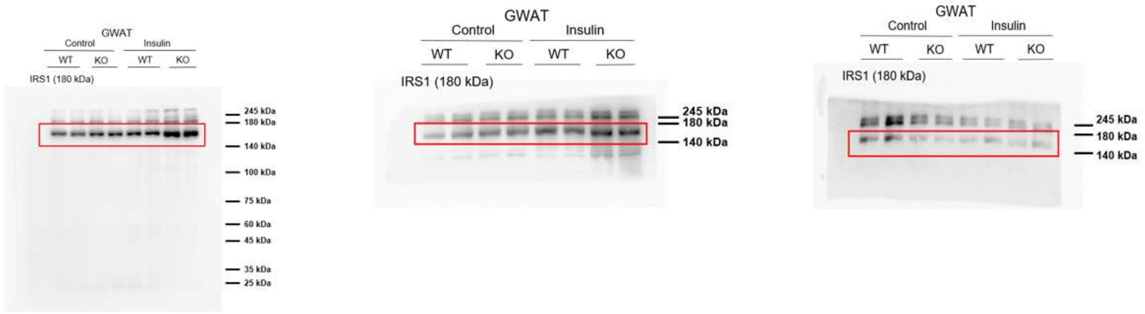

Figure S9I GWAT

Tubulin

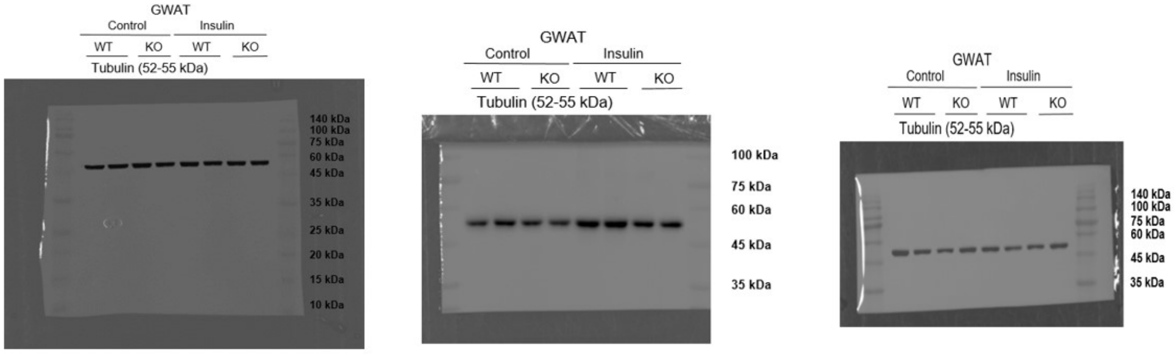

Figure S12B

TM4SF19

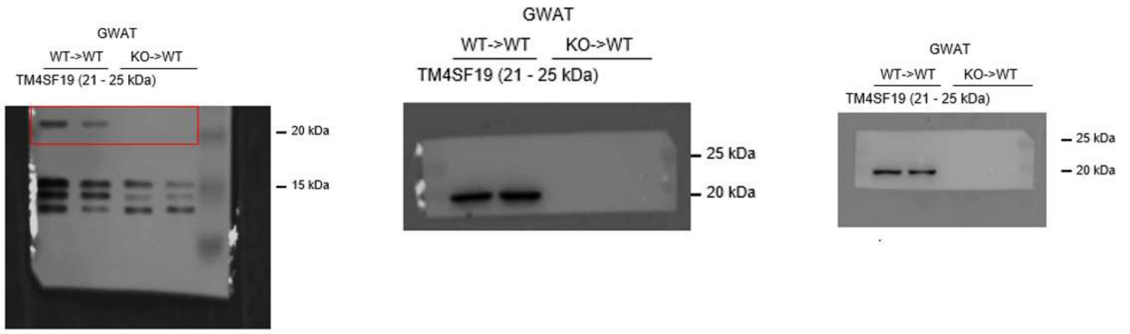

Tubulin

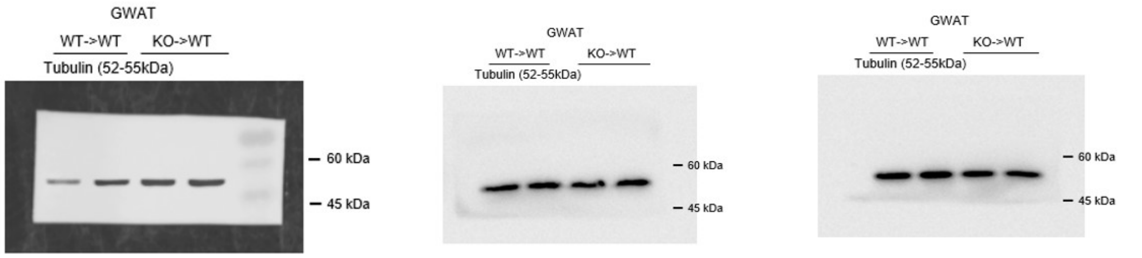

Supplement: Supplementary file 1 — Supplementary Information [file 41467_2024_47108_MOESM1_ESM.pdf]
