# Supplementary material for: TM4SF19-mediated control of lysosomal activity in macrophages contributes to obesity-induced inflammation and metabolic dysfunction
Source: Nat Commun. 2024 Mar 30;15:2779. doi: 10.1038/s41467-024-47108-8 (PMC10981689; doi:10.1038/s41467-024-47108-8)
Supplement: Supplementary file 3 — Description of Additional Supplementary Files [file 41467_2024_47108_MOESM3_ESM.pdf]

Description of Additional Supplementary Files

Supplementary Movie 1: Two-photon intravital imaging of adipose tissue in WT and TM4SF19 KO mice: Two-photon intravital imaging of Cx3Cr1-GFP+ macrophages in WT and TM4SF19 KO mice. PDGFRA-tdTomato-reporter expression visualized PDGFRA+ cells and PDGFRA+ progenitor-derived adipocytes (related to Figure 5H).

Supplementary Movie 2: Two-photon intravital imaging of adipose tissue in WT and TM4SF19 KO mice, showing phagocytosis of adipocyte by macrophages: Two-photon intravital imaging of Cx3Cr1-GFP+ macrophages in WT and TM4SF19 KO mice. CX3CR1-GFP (green) and WGA-blue (red in Figure) visualized phagocytes (related to Figure 5J).
