# Supplementary material for: TM4SF19-mediated control of lysosomal activity in macrophages contributes to obesity-induced inflammation and metabolic dysfunction
Source: Nat Commun. 2024 Mar 30;15:2779. doi: 10.1038/s41467-024-47108-8 (PMC10981689; doi:10.1038/s41467-024-47108-8)
Supplement: Supplementary file 6 — Reporting Summary [file 41467_2024_47108_MOESM6_ESM.pdf]

nature portfolio

Corresponding author(s): Yun-Hee Lee

Last updated by author(s): Feb 21, 2024

Reporting Summary

Nature Portfolio wishes to improve the reproducibility of the work that we publish. This form provides structure for consistency and transparency in reporting. For further information on Nature Portfolio policies, see our Editorial Policies and the Editorial Policy Checklist.

Statistics

For all statistical analyses, confirm that the following items are present in the figure legend, table legend, main text, or Methods section.

n/a	Confirmed	
☐	☒	The exact sample size (*n*) for each experimental group/condition, given as a discrete number and unit of measurement
☐	☒	A statement on whether measurements were taken from distinct samples or whether the same sample was measured repeatedly
☐	☒	The statistical test(s) used AND whether they are one- or two-sided<br>*Only common tests should be described solely by name; describe more complex techniques in the Methods section.*
☒	☐	A description of all covariates tested
☒	☐	A description of any assumptions or corrections, such as tests of normality and adjustment for multiple comparisons
☐	☒	A full description of the statistical parameters including central tendency (e.g. means) or other basic estimates (e.g. regression coefficient) AND variation (e.g. standard deviation) or associated estimates of uncertainty (e.g. confidence intervals)
☐	☒	For null hypothesis testing, the test statistic (e.g. *F*, *t*, *r*) with confidence intervals, effect sizes, degrees of freedom and *P* value noted<br>*Give P values as exact values whenever suitable.*
☒	☐	For Bayesian analysis, information on the choice of priors and Markov chain Monte Carlo settings
☒	☐	For hierarchical and complex designs, identification of the appropriate level for tests and full reporting of outcomes
☐	☒	Estimates of effect sizes (e.g. Cohen's *d*, Pearson's *r*), indicating how they were calculated

Our web collection on statistics for biologists contains articles on many of the points above.

Software and code

Policy information about availability of computer code

Data collection	EvolutionCapt software (ver.17.03) was used for capturing Immunoblot images. Bio-Rad CFX Maestro software (ver.11.2) was used to acquire qPCR data. Nikon Elements (NIS BR Analysis ver. 5.10.00), Zeiss Zen software (ZEN 2.3 Blue edition), Leica LAS X software (Leica Application Suite X 3.5.5. 19976) were used to acquire microscopy images. BD FACSDiva software (ver.9.0) was applied to acquire flow cytometry data. Skanit software ver 5.0 was used to measure the absorbance for BCA assay. Volocity (v7.0), Imaris software(v7.2.3), and MATLAB software(v7.3) were used to acquire the data of two-photon intravital imaging of adipose tissue.
Data analysis	GraphPad Prism9 software was used for statistical test and visualizing graph.NIH ImageJ software was used to quantify Immunoblot images. FlowJo software ver 10.6.1 was applied to analyze flow cytometry data.Heatmap was generated by Morpheus program.Raw reads from snRNA-seq were aligned to the mouse genome (mm10) using cellranger (ver 6.1.1). snRNA-seq data analysis was performed in R (ver 4.1.0) and python (ver 3.10.0). scater (ver 1.22.0) was used for quality control and scDblFinder (ver 1.8.0) was used to identify putative doublets, scran (ver 1.22.1) was used for normalization and selecting highly variable genes. Seurat (ver 4.0.5) was used for clustering, dimension reduction, gene expression analysis and calculating module scores, harmony (ver 0.1.0) was used to correct batch effect during annotation process. Palantir (ver 1.0.0) was used for trajectory analysis and Monocle3 (ver 1.3.1) was used to confirm the trajectory, limma (ver 3.50.0) was used for differential expression analysis and fgsea (ver 1.21.2) was used for gene set enrichment analysis using Molecular Signature Database (MSigDB) downloaded using msigdbr (ver 7.4.1). MASC (ver 0.0.0.9000) was used to determine significance of cell-type compositional change. hdWGCNA (ver 0.2.04) was used to construct co-expression gene modules. SCORPIUS (ver 1.0.9) was used to determine trajectory of Trem2- to Trem2+ macrophages.

For manuscripts utilizing custom algorithms or software that are central to the research but not yet described in published literature, software must be made available to editors and reviewers. We strongly encourage code deposition in a community repository (e.g. GitHub). See the Nature Portfolio guidelines for submitting code & software for further information.

Data

Policy information about availability of data

All manuscripts must include a data availability statement. This statement should provide the following information, where applicable:

- Accession codes, unique identifiers, or web links for publicly available datasets
- A description of any restrictions on data availability
- For clinical datasets or third party data, please ensure that the statement adheres to our policy

The raw snRNA-seq data generated in this study have been deposited in the NCBI Sequence Read Archive (SRA) database under accession code PRJNA942977 [https://www.ncbi.nlm.nih.gov/bioproject/PRJNA942977]. The publicly available human and mouse adipose tissue data used in this study are available in the gene expression omnibus (GEO) database under accession code GSE59034[https://www.ncbi.nlm.nih.gov/geo/query/acc.cgi?acc=GSE59034], GSE150102[https://www.ncbi.nlm.nih.gov/geo/query/acc.cgi?acc=GSE150102] and GSE182930[https://www.ncbi.nlm.nih.gov/geo/query/acc.cgi?acc=GSE182930]. The publicly available human white adipose tissue sc/snRNA-seq data is available under accession code GSE176171 [https://www.ncbi.nlm.nih.gov/geo/query/acc.cgi?acc=GSE176171]. Mouse reference genome GRCm38 (mm10) was used to align raw reads. All data generated in this study are provided in the Article, Supplementary Information, and Source Data file. Source Data are provided with this paper. Other data supporting the findings of this study are available from the corresponding authors on request.

Research involving human participants, their data, or biological material

Policy information about studies with human participants or human data. See also policy information about sex, gender (identity/presentation), and sexual orientation and race, ethnicity and racism.

Reporting on sex and gender	For human samples, there were 15 male and 9 female participants. The characteristics of human fat tissues from 24 individuals are available in Supplementary Table 2. Participants of both sexes volunteered for this study, and we did not intend to selectively recruit a particular gender. The sex/gender of participants was determined through self-report.
Reporting on race, ethnicity, or other socially relevant groupings	All subjects in this study are Asian.
Population characteristics	9~79 years old participants were included in this study. The range of BMI from participants was 19.11 ~ 32.38 kg/m^2. Their heights were included in 136.8 ~ 178. The range of body weight from all participants were 40.8 ~ 91.2. The detailed information of participants are shown in the Supplementary Table 2.
Recruitment	Subjects were patients admitted to Korea University Guro Hospital and voluntarily participated in this study. Participants were recruited randomly as a part of the protocol ((IRB no.:2022GR0095) without patient self-selection bias to impact the results in this paper. Human subcutaneous fat tissues were collected from patients undergoing benign mass removal surgery at the Department of Plastic Surgery of the Korea University Guro Hospital (KUGH), Seoul, Republic of Korea from January 2022 to April 2022 and tissues.
Ethics oversight	All participants provided written informed consent, and ethical approval for this study was granted by the Ethical Committee of Korea University Guro Hospital. The study was conducted in full accordance with the principles of the Declaration of Helsinki.

Note that full information on the approval of the study protocol must also be provided in the manuscript.

Field-specific reporting

Please select the one below that is the best fit for your research. If you are not sure, read the appropriate sections before making your selection.

☒ Life sciences ☐ Behavioural & social sciences ☐ Ecological, evolutionary & environmental sciences

For a reference copy of the document with all sections, see nature.com/documents/nr-reporting-summary-flat.pdf

Life sciences study design

All studies must disclose on these points even when the disclosure is negative.

Sample size	No statistical method was performed to predetermine sample size. All in vivo studies included a sample size of at least six mice per group, which was typically sufficient to determine statistical significance between groups, according to our prior experience and community standards. For the intravital imaging experiments by using two-photon microscopy, three biologically independent animals were used for each condition. All data from in vitro experiments were performed in at least three independent experiments.
Data exclusions	No data were intentionally excluded from the analyses.
Replication	All data show the means ± standard error of the mean (SEM) of at least three biological replicates with the n indicated in each experiment. All experiments were carried out under standard and clearly defined conditions and all attempts at replication were successful. For

	reproducibility of snRNA-seq data, two biological replicates were used for each condition.
Randomization	All in vitro and in vivo experiments were conducted with randomization procedures. In the in vivo experiments, age-matched animals were randomized into groups at the beginning of the study. In the case of in vitro experiments, individual wells were randomly assigned to different experimental conditions.
Blinding	Investigators maintained blinding throughout all in vivo and in vitro experiments, including the following analyses: phagocytosis assay, lysosomal activity assessment, body weight monitoring, indirect calorimetry, plasma glucose measurement, immunohistochemistry, and histological analysis. The metadata information was not blinded for the snRNA-seq analyses. Given that this analysis investigates alterations in cell type composition and transcriptome under varying conditions, blinding was irrelevant for this aspect of the study.

Behavioural & social sciences study design

All studies must disclose on these points even when the disclosure is negative.

Study description	*Briefly describe the study type including whether data are quantitative, qualitative, or mixed-methods (e.g. qualitative cross-sectional, quantitative experimental, mixed-methods case study).*
Research sample	*State the research sample (e.g. Harvard university undergraduates, villagers in rural India) and provide relevant demographic information (e.g. age, sex) and indicate whether the sample is representative. Provide a rationale for the study sample chosen. For studies involving existing datasets, please describe the dataset and source.*
Sampling strategy	*Describe the sampling procedure (e.g. random, snowball, stratified, convenience). Describe the statistical methods that were used to predetermine sample size OR if no sample-size calculation was performed, describe how sample sizes were chosen and provide a rationale for why these sample sizes are sufficient. For qualitative data, please indicate whether data saturation was considered, and what criteria were used to decide that no further sampling was needed.*
Data collection	*Provide details about the data collection procedure, including the instruments or devices used to record the data (e.g. pen and paper, computer, eye tracker, video or audio equipment) whether anyone was present besides the participant(s) and the researcher, and whether the researcher was blind to experimental condition and/or the study hypothesis during data collection.*
Timing	*Indicate the start and stop dates of data collection. If there is a gap between collection periods, state the dates for each sample cohort.*
Data exclusions	*If no data were excluded from the analyses, state so OR if data were excluded, provide the exact number of exclusions and the rationale behind them, indicating whether exclusion criteria were pre-established.*
Non-participation	*State how many participants dropped out/declined participation and the reason(s) given OR provide response rate OR state that no participants dropped out/declined participation.*
Randomization	*If participants were not allocated into experimental groups, state so OR describe how participants were allocated to groups, and if allocation was not random, describe how covariates were controlled.*

Ecological, evolutionary & environmental sciences study design

All studies must disclose on these points even when the disclosure is negative.

Study description	*Briefly describe the study. For quantitative data include treatment factors and interactions, design structure (e.g. factorial, nested, hierarchical), nature and number of experimental units and replicates.*
Research sample	*Describe the research sample (e.g. a group of tagged Passer domesticus, all Stenocereus thurberi within Organ Pipe Cactus National Monument), and provide a rationale for the sample choice. When relevant, describe the organism taxa, source, sex, age range and any manipulations. State what population the sample is meant to represent when applicable. For studies involving existing datasets, describe the data and its source.*
Sampling strategy	*Note the sampling procedure. Describe the statistical methods that were used to predetermine sample size OR if no sample-size calculation was performed, describe how sample sizes were chosen and provide a rationale for why these sample sizes are sufficient.*
Data collection	*Describe the data collection procedure, including who recorded the data and how.*
Timing and spatial scale	*Indicate the start and stop dates of data collection, noting the frequency and periodicity of sampling and providing a rationale for these choices. If there is a gap between collection periods, state the dates for each sample cohort. Specify the spatial scale from which the data are taken*
Data exclusions	*If no data were excluded from the analyses, state so OR if data were excluded, describe the exclusions and the rationale behind them, indicating whether exclusion criteria were pre-established.*
Reproducibility	*Describe the measures taken to verify the reproducibility of experimental findings. For each experiment, note whether any attempts to*

Reproducibility	*repeat the experiment failed OR state that all attempts to repeat the experiment were successful.*
Randomization	*Describe how samples/organisms/participants were allocated into groups. If allocation was not random, describe how covariates were controlled. If this is not relevant to your study, explain why.*
Blinding	*Describe the extent of blinding used during data acquisition and analysis. If blinding was not possible, describe why OR explain why blinding was not relevant to your study.*

Did the study involve field work? ☐ Yes ☒ No

Reporting for specific materials, systems and methods

We require information from authors about some types of materials, experimental systems and methods used in many studies. Here, indicate whether each material, system or method listed is relevant to your study. If you are not sure if a list item applies to your research, read the appropriate section before selecting a response.

Materials & experimental systems

n/a	Involved in the study	
☐	☒	Antibodies
☐	☒	Eukaryotic cell lines
☒	☐	Palaeontology and archaeology
☐	☒	Animals and other organisms
☒	☐	Clinical data
☒	☐	Dual use research of concern
☒	☐	Plants

Methods

n/a	Involved in the study	
☒	☐	ChIP-seq
☐	☒	Flow cytometry
☒	☐	MRI-based neuroimaging

Antibodies

Antibodies used

Anti-rabbit TM4SF19 antibody (Immunoway, YT6290, WB 1:1000)
Anti-rabbit a/P-Tubulin (Cell Signaling, 2148 ,WB 1:2000)
Anti-rabbit NF-kB pl05/50 (Cell Signaling, 13586 ,WB 1:1000)
Anti-rabbit ATP6V1B2 (Cell Signaling, 14617, WB 1:1000)
Anti-rabbit LAMP1 (Cell Signaling, 3243, WB 1:1000, IHC 1:200)
Anti-rabbit F4/80 (Cell Signaling, 30325, IHC 1:200)
Anti-rabbit p-IRSl (S612) (Cell Signaling,3203, WB 1:1000)
Anti-rabbit I RSI (Cell Signaling, 3407, WB 1:1000)
Anti-rabbit COXIV (Cell Signaling, 4850, WB 1:1000)
Anti-rabbit Myc-Tag (Cell Signaling, 2278, IP 1:200)
Normal rabbit IgG (Cell Signaling, 2729, IP 1:100)
Anti-rabbit ATP6V0B (Novus Biological, NBP2-83943 WB 1:500)
Anti-rabbit ATP6V1A (GeneTEX, GTX110815, WB 1:1000)
Anti-mouse p-AKT (S473) (Santa Cruz, Sc-514032, WB (1:1000))
Anti-mouse AKT (Santa Cruz, Sc-81434, WB 1:1000)
Anti-mouse TOTAL OXPHOS cocktail (Abcam, AbllO413,WB 1:1000)
Anti-mouse ATP6V0D2 (Santa Cruz, Sc-517031, WB 1:1000)
Anti-rabbit PDI (Cell Signaling, 3501, IHC 1:200)
PE anti-mouse/human LYVE1 (Invitrogen, 12-0443-82)
PE/Cyanine7 anti-mouse CD11C (Biolegend,117317)
BV421 anti-mouse CD206 (Biolegend, 141717)
BV711 anti-mouse CD11b (Biolegend, 101242)
FITC anti-mouse CD11b (Biolegend, 101206)
Human/Mouse TREM2 Allophycocyanin MAb (R&D systems FAB17291A)
APC anti-mouse CD45 (Biolegend, 103116)
FITC anti-mouse CD45 (Invitrogen, 11-0451-82)

Validation

All these antibodies were validated by manufacturers (please see the datasheet using the Cat#). Moreover, the total OXPHOS rodent WB antibody cocktail (Abeam #AbllO413) are routinely used for mitochondrial ETC immunoblotting.

Anti-rabbit TM4SF19 antibody (Immunoway, YT6290)
http://www.immunoway.com/Home/22/YT6290
Host Organism: rabbit
Clonality: polyclonal
Dilution: WB 1:1000

Anti-rabbit a/b-Tubulin (Cell Signaling, 2148)
http://www.cellsignal.com/products/primary-antibodies/a-b-tubulin-antibody/2148
Host Organism: rabbit

Clonality: polyclonal
Dilution: WB 1:2000

Anti-rabbit NF-kB p105/50 (Cell Signaling, 13586)
https://www.cellsignal.com/products/primary-antibodies/nf-kb1-p105-p50-d4p4d-rabbit-mab/13586
Host Organism: rabbit
Clonality: polyclonal
Dilution: WB 1:1000

Anti-rabbit ATP6V1B2 (Cell Signaling, 14617)
https://www.cellsignal.com/products/primary-antibodies/atp6v1b2-d2f9r-rabbit-mab/14617
Host Organism: rabbit
Clonality: polyclonal
Dilution: WB 1:1000

Anti-rabbit LAMP1 (Cell Signaling, 3243)
https://www.cellsignal.com/products/primary-antibodies/lamp1-c54h11-rabbit-mab/3243
Host Organism: rabbit
Clonality: polyclonal
Dilution: WB 1:1000, IHC 1:200

Anti-rabbit F4/80 (Cell Signaling, 30325)
https://www.cellsignal.com/products/primary-antibodies/f4-80-d4c8v-xp-rabbit-mab/30325
Host Organism: rabbit
Clonality: polyclonal
Dilution: IHC 1:200

Anti-rabbit p-IRS1 (S612) (Cell Signaling,3203)
https://www.cellsignal.com/products/primary-antibodies/phospho-irs-1-ser612-c15h5-rabbit-mab/3203
Host Organism: rabbit
Clonality: polyclonal
Dilution: WB 1:1000

Anti-rabbit IRS1 (Cell Signaling, 3407)
https://www.cellsignal.com/products/primary-antibodies/irs-1-d23g12-rabbit-mab/3407
Host Organism: rabbit
Clonality: polyclonal
Dilution: WB 1:1000

Anti-rabbit COXIV (Cell Signaling, 4850)
https://www.cellsignal.com/products/primary-antibodies/cox-iv-3e11-rabbit-mab/4850
Host Organism: rabbit
Clonality: polyclonal
Dilution: WB 1:1000

Anti-rabbit Myc-Tag (Cell Signaling, 2278)
https://www.cellsignal.com/products/primary-antibodies/myc-tag-71d10-rabbit-mab/2278
Host Organism: rabbit
Clonality: polyclonal
Dilution: IP 1:200

Normal rabbit IgG (Cell Signaling, 2729)
https://www.cellsignal.com/products/primary-antibodies/normal-rabbit-igg/2729
Host Organism: rabbit
Clonality: polyclonal
Dilution: IP 1:100

Anti-rabbit ATP6V0B (Novus Biological, NBP2-83943)
https://www.novusbio.com/products/atp6v0b-antibody_nbp2-83943#datasheet
Host Organism: rabbit
Clonality: polyclonal
Dilution: WB 1:500

Anti-rabbit ATP6V1A (GeneTEX, GTX110815)
https://www.genetex.com/Product/Detail/ATP6V1A-antibody/GTX110815
Host Organism: rabbit
Clonality: polyclonal
Dilution: WB 1:1000

Anti-mouse p-AKT (S473) (Santa Cruz, Sc-514032)
https://www.scbt.com/ko/p/p-akt1-2-3-antibody-c-11
Host Organism: mouse
Clonality: monoclonal
Dilution: WB 1:1000

Anti-mouse AKT (Santa Cruz, Sc-81434)

https://www.scbt.com/p/akt1-2-3-antibody-5c10
Host Organism: mouse
Clonality: monoclonal
Dilution: WB 1:1000

Anti-mouse TOTAL OXPHOS cocktail (Abcam, Ab110413)
https://www.abcam.com/en-kr/products/panels/total-oxphos-rodent-wb-antibody-cocktail-ab110413
Host Organism: mouse
Clonality: monoclonal
Dilution: WB 1:1000

Anti-mouse ATP6V0D2 (Santa Cruz, Sc-517031)
https://www.scbt.com/ko/p/v-atpase-d2-antibody-7a4
Host Organism: mouse
Clonality: monoclonal
Dilution: WB 1:1000

Anti-rabbit PDI (Cell Signaling, 3501)
https://www.cellsignal.com/products/primary-antibodies/pdi-c81h6-rabbit-mab/3501
Host Organism: rabbit
Clonality: polyclonal
Dilution: IHC 1:200

FITC anti-mouse CD45 (Invitrogen, 11-0451-82)
https://www.thermofisher.com/antibody/product/CD45-Antibody-clone-30-F11-Monoclonal/11-0451-82
Host Organism: Rat
Clonality: monoclonal
Dilution: FACS 1:200

APC anti-mouse CD45 (Biolegend, 103116)
https://www.biolegend.com/ja-jp/products/apc-cyanine7-anti-mouse-cd45-antibody-2530?GroupID=BLG1932
Host Organism: Rat
Clonality: monoclonal
Dilution: FACS 1:200

Human/Mouse TREM2 Allophycocyanin MAb (R&D systems FAB17291A)
https://www.rndsystems.com/products/human-mouse-trem2-apc-conjugated-antibody-237920_fab17291a
Host Organism: Rat
Clonality: monoclonal
Dilution: FACS 1:100

FITC anti-mouse CD11b (Biolegend, 101206)
https://www.biolegend.com/de-at/products/fitc-anti-mouse-human-cd11b-antibody-347?GroupID=BLG10660
Host Organism: Rat
Clonality: monoclonal
Dilution: FACS 1:100

BV711 anti-mouse CD11b (Biolegend, 101242)
https://www.biolegend.com/fr-fr/products/brilliant-violet-711-anti-mouse-human-cd11b-antibody-7927?GroupID=BLG10552
Host Organism: Rat
Clonality: monoclonal
Dilution: FACS 1:100

BV421 anti-mouse CD206 (Biolegend, 141717)
https://www.biolegend.com/en-gb/productstab/brilliant-violet-421-anti-mouse-cd206-mmr-antibody-8638
Host Organism: Rat
Clonality: monoclonal
Dilution: FACS 1:100

PE/Cyanine7 anti-mouse CD11C (Biolegend, 117317)
https://www.biolegend.com/en-gb/products/pe-cyanine7-anti-mouse-cd11c-antibody-3086?GroupID=BLG11937
Host Organism: Rat
Clonality: monoclonal
Dilution: FACS 1:100

PE anti-mouse/human LYVE1 (Invitrogen, 12-0443-82)
https://thermofisher.com/antibody/product/LYVE1-Antibody-clone-ALY7-Monoclonal/12-0443-82
Host Organism: Rat
Clonality: monoclonal
Dilution: FACS 1:50

Eukaryotic cell lines

Policy information about cell lines and Sex and Gender in Research

Cell line source(s)	HEK293T cells (human, ATCC CRL-3216)<br>RAW264.7 cells (mouse, ATCC TIB-71)<br>C3H10T1/2 cells (mouse, ATCC CCL-226)
Authentication	HEK293T and RAW264.7 cells had been authenticated by the provider (ATCC) using STR profiling. We further authenticated the cell line by examining the morphological characteristics of the cell line by microscope. C3H10T1/2 cells were obtained from ATCC and the adipogenic identity of C3H10T1/2 cell line has been validated in the lab by its morphological characteristics and lipid staining via microscope.
Mycoplasma contamination	The cell lines were determined to be free from Mycoplasma contamination.<br>No indication of contamination was observed during experiments.
Commonly misidentified lines<br>(See ICLAC register)	No commonly misidentified cell lines were used.

Palaeontology and Archaeology

Specimen provenance	N/A
Specimen deposition	N/A
Dating methods	N/A

☐ Tick this box to confirm that the raw and calibrated dates are available in the paper or in Supplementary Information.

Ethics oversight	N/A

Note that full information on the approval of the study protocol must also be provided in the manuscript.

Animals and other research organisms

Policy information about studies involving animals; ARRIVE guidelines recommended for reporting animal research, and Sex and Gender in Research

Laboratory animals

All mice used in the experiments for this study were male, aged between 6 and 16 weeks old. Mice were housed in a temperature- and humidity-controlled, specific pathogen-free animal facility at 22± 1°C, under a 12:12 h light:dark cycle, and health status checks were performed two or three times a week.
1. Mouse, C57BL/6N-Tm4sfl9emlcyagen
In TM4SF19 whole body knock-out mouse, exon 2-5 of the Tm4sf19 gene were deleted. mice were obtained from Cyagen.

2. Mouse, C57BL/6JSmoc-Tm4sfl9eml(flox)Smoc
In this strain, loxP sites flanking exon 2-3 of Tm4sf19 gene were carried. Mice were obtained from Shanghai model organism.

3. Mouse, FVB-Tg (Csflr-cre/Esrl*)lJwp/J
These mice express the tamoxifen-inducible MerCreMer fusion protein under control of the Csf1r promoter. Mice were obtained from The Jackson Laboratory.

4. Mouse, B6.Cg-Gt(ROSA)26Sortm9(CAG-tdTomato)Hze 651 /J
Mice express robust tdTomato fluorescence following Cre-mediated recombination. Mice were obtained from The Jackson Laboratory.

5. Mouse, C57BL/6-Tg(Pdgfra-cre)1Clc/J
These mice expresses Cre recombinase expression by endogenous promoter/enhancer elements of the Pdgfra gene. Mice were obtained from The Jackson Laboratory.

6. Mouse, B6.129P2(Cg)-Cx3cr1tm1Litt 652 /J
These mice express EGFP in monocytes, dendritic cells, NK cells, and brain microglia under control of the endogenous Cx3cr1 locus.
Mice were obtained form The Jackson Laboratory.

Wild animals	No wild animals were used in this study.

Reporting on sex	This study was performed on male mice.
Field-collected samples	No field-collected samples were used in this study.
Ethics oversight	All of protocols related to animal experiment were approved by the Institutional Animal Care and Use Committees of Seoul National University (SNU-211209-4-1, SNU-210403-2, SNU-230130-5).

Note that full information on the approval of the study protocol must also be provided in the manuscript.

Clinical data

Policy information about clinical studies

All manuscripts should comply with the ICMJE guidelines for publication of clinical research and a completed CONSORT checklist must be included with all submissions.

Clinical trial registration	N/A
Study protocol	The study protocol was approved by the Institutional Review Board of the authors' institution (IRB No. 2022GR0095). This study was conducted in accordance with the Declaration of Helsinki, and informed consent was obtained from each patient.
Data collection	Human subcutaneous fat tissues were collected from the Department of Plastic Surgery of Korea University Guro Hospital, Seoul, Republic of Korea, between January 2022 and April 2022. The tissues were stored at -80°C. The characteristics of human fat tissues from 24 individuals are available in Supplementary Table 2.
Outcomes	N/A

Dual use research of concern

Policy information about dual use research of concern

Hazards

Could the accidental, deliberate or reckless misuse of agents or technologies generated in the work, or the application of information presented in the manuscript, pose a threat to:

No	Yes	
☐	☐	Public health
☐	☐	National security
☐	☐	Crops and/or livestock
☐	☐	Ecosystems
☐	☐	Any other significant area

Experiments of concern

Does the work involve any of these experiments of concern:

No	Yes	
☐	☐	Demonstrate how to render a vaccine ineffective
☐	☐	Confer resistance to therapeutically useful antibiotics or antiviral agents
☐	☐	Enhance the virulence of a pathogen or render a nonpathogen virulent
☐	☐	Increase transmissibility of a pathogen
☐	☐	Alter the host range of a pathogen
☐	☐	Enable evasion of diagnostic/detection modalities
☐	☐	Enable the weaponization of a biological agent or toxin
☐	☐	Any other potentially harmful combination of experiments and agents

Plants

Seed stocks | N/A

Novel plant genotypes | N/A

Authentication | N/A

ChIP-seq

Data deposition

☐ Confirm that both raw and final processed data have been deposited in a public database such as GEO.

☐ Confirm that you have deposited or provided access to graph files (e.g. BED files) for the called peaks.

Data access links
May remain private before publication. | *For "Initial submission" or "Revised version" documents, provide reviewer access links. For your "Final submission" document, provide a link to the deposited data.*

Files in database submission | *Provide a list of all files available in the database submission.*

Genome browser session
(e.g. UCSC) | *Provide a link to an anonymized genome browser session for "Initial submission" and "Revised version" documents only, to enable peer review. Write "no longer applicable" for "Final submission" documents.*

Methodology

Replicates | *Describe the experimental replicates, specifying number, type and replicate agreement.*

Sequencing depth | *Describe the sequencing depth for each experiment, providing the total number of reads, uniquely mapped reads, length of reads and whether they were paired- or single-end.*

Antibodies | *Describe the antibodies used for the ChIP-seq experiments; as applicable, provide supplier name, catalog number, clone name, and lot number.*

Peak calling parameters | *Specify the command line program and parameters used for read mapping and peak calling, including the ChIP, control and index files used.*

Data quality | *Describe the methods used to ensure data quality in full detail, including how many peaks are at FDR 5% and above 5-fold enrichment.*

Software | *Describe the software used to collect and analyze the ChIP-seq data. For custom code that has been deposited into a community repository, provide accession details.*

Flow Cytometry

Plots

Confirm that:

☒ The axis labels state the marker and fluorochrome used (e.g. CD4-FITC).

☒ The axis scales are clearly visible. Include numbers along axes only for bottom left plot of group (a 'group' is an analysis of identical markers).

☒ All plots are contour plots with outliers or pseudocolor plots.

☒ A numerical value for number of cells or percentage (with statistics) is provided.

Methodology

Sample preparation | Gonadal white adipose tissues were dissected, minced, and digested with collagenase type I in KRBB containing 3% BSA at 37°C. Stroma vascular cells (SVC) were washed with KRBB buffer containing 3% BSA. And SVC were stained with fluorescence labeled primary antibodies for 20min at room temperature.

Instrument | LSRFortessa X-20 Flow Cytometer (BD Biosciences) was used for flow cytometry.

Software | BD FACSDiva 9.0 software was applied to acquire flow cytometry data. FlowJo software version 10.6.1 was applied to analyze flow cytometry data

Cell population abundance | Populations were identified as described in the Methods and Supplemental materials. Gating Strategy is described below.

Gating strategy | The gating strategy for sorting is detailed in the Supplemental Information.
FSC-A vs. SSC-A gating was used to identify the distinct cells and to exclude debris.
Singlets were identified by FSC-H vs. FSC-H gating.
Macrophages were identified by CD45 and CD11b or F4/80.

☒ Tick this box to confirm that a figure exemplifying the gating strategy is provided in the Supplementary Information.

Magnetic resonance imaging

Experimental design

Design type | *Indicate task or resting state; event-related or block design.*

Design specifications | *Specify the number of blocks, trials or experimental units per session and/or subject, and specify the length of each trial or block (if trials are blocked) and interval between trials.*

Behavioral performance measures | *State number and/or type of variables recorded (e.g. correct button press, response time) and what statistics were used to establish that the subjects were performing the task as expected (e.g. mean, range, and/or standard deviation across subjects).*

Acquisition

Imaging type(s) | *Specify: functional, structural, diffusion, perfusion.*

Field strength | *Specify in Tesla*

Sequence & imaging parameters | *Specify the pulse sequence type (gradient echo, spin echo, etc.), imaging type (EPI, spiral, etc.), field of view, matrix size, slice thickness, orientation and TE/TR/flip angle.*

Area of acquisition | *State whether a whole brain scan was used OR define the area of acquisition, describing how the region was determined.*

Diffusion MRI ☐ Used ☐ Not used

Preprocessing

Preprocessing software | *Provide detail on software version and revision number and on specific parameters (model/functions, brain extraction, segmentation, smoothing kernel size, etc.).*

Normalization | *If data were normalized/standardized, describe the approach(es): specify linear or non-linear and define image types used for transformation OR indicate that data were not normalized and explain rationale for lack of normalization.*

Normalization template | *Describe the template used for normalization/transformation, specifying subject space or group standardized space (e.g. original Talairach, MNI305, ICBM152) OR indicate that the data were not normalized.*

Noise and artifact removal | *Describe your procedure(s) for artifact and structured noise removal, specifying motion parameters, tissue signals and physiological signals (heart rate, respiration).*

Volume censoring | *Define your software and/or method and criteria for volume censoring, and state the extent of such censoring.*

Statistical modeling & inference

Model type and settings | *Specify type (mass univariate, multivariate, RSA, predictive, etc.) and describe essential details of the model at the first and second levels (e.g. fixed, random or mixed effects; drift or auto-correlation).*

Effect(s) tested | *Define precise effect in terms of the task or stimulus conditions instead of psychological concepts and indicate whether ANOVA or factorial designs were used.*

Specify type of analysis: ☐ Whole brain ☐ ROI-based ☐ Both

Statistic type for inference
(See Eklund et al. 2016) | *Specify voxel-wise or cluster-wise and report all relevant parameters for cluster-wise methods.*

Correction | *Describe the type of correction and how it is obtained for multiple comparisons (e.g. FWE, FDR, permutation or Monte Carlo).*

Models & analysis

n/a	Involved in the study
☐	☐ Functional and/or effective connectivity
☐	☐ Graph analysis
☐	☐ Multivariate modeling or predictive analysis

Functional and/or effective connectivity	*Report the measures of dependence used and the model details (e.g. Pearson correlation, partial correlation, mutual information).*
Graph analysis	*Report the dependent variable and connectivity measure, specifying weighted graph or binarized graph, subject- or group-level, and the global and/or node summaries used (e.g. clustering coefficient, efficiency, etc.).*
Multivariate modeling and predictive analysis	*Specify independent variables, features extraction and dimension reduction, model, training and evaluation metrics.*
